# Partitioning stable and unstable expression level variation in cell populations: A theoretical framework and its application to the T cell receptor

**DOI:** 10.1371/journal.pcbi.1007910

**Published:** 2020-08-25

**Authors:** Thiago S. Guzella, Vasco M. Barreto, Jorge Carneiro

**Affiliations:** 1 Instituto Gulbenkian de Ciência, Oeiras, Portugal; 2 CEDOC - Chronic Diseases Research Center, NOVA Medical School, Universidade Nova de Lisboa, Lisboa, Portugal; National Institutes of Health, UNITED STATES

## Abstract

Phenotypic variation in the copy number of gene products expressed by cells or tissues has been the focus of intense investigation. To what extent the observed differences in cellular expression levels are persistent or transient is an intriguing question. Here, we develop a quantitative framework that resolves the expression variation into stable and unstable components. The difference between the expression means in two cohorts isolated from any cell population is shown to converge to an asymptotic value, with a characteristic time, *τ*_*T*_, that measures the timescale of the unstable dynamics. The asymptotic difference in the means, relative to the initial value, measures the stable proportion of the original population variance $R_{\alpha }^{2}$. Empowered by this insight, we analysed the T-cell receptor (TCR) expression variation in CD4 T cells. About 70% of TCR expression variance is stable in a diverse polyclonal population, while over 80% of the variance in an isogenic TCR transgenic population is volatile. In both populations the TCR levels fluctuate with a characteristic time of 32 hours. This systematic characterisation of the expression variation dynamics, relying on time series of cohorts’ means, can be combined with technologies that measure gene or protein expression in single cells or in bulk.

## Author summary

No two cells are identical. Even isogenic cells, living in the same environment and expressing the same set of genes display measurable differences or variation in the expression level of any of these genes. How much of the differences in expression levels are permanent and how much of these differences vanish in time has intrigued us for generations. We develop a theoretical framework based on a stochastic model and put it to work in the analysis of T cell receptor expression level in CD4 T cells. We show that T cell populations with genetically diverse receptors display stable variation in receptor expression but, surprisingly, we detect persistent differences in receptor levels among uniform transgenic T cells. The analysis, being based on the mean cohort expression levels logarithm, can be applied to techniques that measure expression at single-cell level and also to the myriad of genomics and proteomics techniques that measure expression in bulk populations.

## Introduction

The phenotypic variation among organisms or cells is a theme of growing importance in biology. Macroscopic phenotypes, such as body structures or physiologic responses, have been studied for ages, but one phenotype particularly suitable for quantification that has received attention in the last decades is the amount of specific mRNAs and proteins expressed by single cells. Advances in genomics have allowed the analysis of genetic contributions to variation in gene expression, in terms of so-called expression quantitative trait loci (eQTL) [1, 2]. In this case, expression levels, typically assessed via mRNA levels, are treated as quantitative traits, and one is interested in the specific loci underlying variation in expression levels among different individuals. The increasing availability of single-cell resolution genomics, proteomics and metabolomics technologies has enabled molecular biologists to analyse cell lineages and tissues showing that what were previously perceived as homogeneous cell populations are in fact a complex mixture of often transient and interchangeable cellular types and cellular states (see discussion in [3]). In parallel to these studies linking phenotypes to genotype, the literature on stochastic gene expression [4–8], reviewed in [9], has brought to light the variation in expression levels in isogenic cells, even when these are in the same cellular state and in the same environment. The variation is typically attributed to the “noise” resulting from the small copy number of molecules involved in the process.

Several studies addressed the fluctuation dynamics of gene expression levels [10, 11] revealing a complex picture of the variation in isogenic cell populations. The fluctuation timescales range from hours [7, 12], to days [13–15] or weeks [16–18], depending on the cells and on the degree of multimodality of the expression distribution under study. The distinct timescales can be associated with the different mechanisms that may cause the variation in the expression levels of a molecular component of interest in some cell population. However, most quantitative approaches developed up to this date have focused on noise in gene expression as the predominant mechanism explaining the variation observed (for example, [6, 7, 19–24]). It remains unclear to which degree less volatile dynamic processes or even persistent differences contribute to the observed variation in a isogenic cell population. This is particularly relevant in the case of cells from multicellular organisms, due to the robust epigenetic processes that underlie differentiation stages, cell lineages or cell states, but also the intraclonal structure of apparently homogenous populations [25, 26].

A case study of particular interest is the expression of Sca1 in a hematopoietic cell line [16, 17] since it reveals the complexity of variation dynamics and also the difficulties in characterising it experimentally. Chang et al. [16] reported that that biased cohorts of cells tend to restore the histogram of expression levels of Sca1 of the starting population, albeit with very slow dynamics. In principle, complete restoration would be consistent with a lack of stable variants in the population. However, Pina et al. [17] have shown that even after 2 weeks, the reconstitution is incomplete. More importantly, some cells in this population express markers of terminal differentiation, and have limited proliferative capacity [17]. This points to an inherent heterogeneity in the population that may persist in time. An important limitation of these approaches was relying mainly on the juxtaposition of histograms of expression levels in order to compare cell populations, without a rigorous quantification. It is not clear how to analyse such data and because of this the degree to which the original distribution is restored remains uncertain. A quantitative approach that overcomes this impasse is necessary and also important to provide formal concepts on which to ground subsequent studies on the expression levels in cell populations.

Our work lumps the molecular mechanisms regulating expression levels in a cell population into two components, one stable and another unstable. The stable component leads to permanent differences between the expression levels of any two cohorts of cells. The unstable component, on the other hand, represents transient differences in the expression levels of the cohorts that eventually vanish in time. Starting from these definitions, a general model is derived to describe protein expression levels in a population. The relative contribution of the stable component to the expression variation is then defined as a single parameter termed $R_{\alpha }^{2}$. We show theoretically that this parameter can be estimated in an unbiased way by following over time the mean expression in cohorts isolated from the population of interest. This dynamical characterisation of the expression variation is completed by concomitantly estimating the characteristic timescale *τ*_*T*_.

This theoretical result is used to characterise the contributions to variation in the expression levels of the T-cell receptor (TCR) in two biologically relevant CD4 T cell populations. The first population, purified from wild type mice, is composed of clones emerging from the process of V(D)J recombination, each carrying genetically distinct TCR loci. The second is a genetically uniform population isolated from Marilyn TCR-transgenic mice, in which all T cells express the same recombined receptor genes [27]. We find that the stable component is the main contribution in the polyclonal population ($R_{\alpha }^{2}\approx 70\%$), while the unstable component predominates in the Marilyn population ($R_{\alpha }^{2}\approx 20\%$). This suggests that genetic heterogeneity contributes to stable differences in TCR expression levels in T cells, but that there are other mechanisms contributing to persistent expression variation in isogenic populations.

## Results

### A general model for protein expression levels in a cell population

#### Partitioning the contributions to variation in expression levels

We assume that any cell population, hereafter referred to as full population, is a mixture of sub-populations. Each cell belongs to and remains in one of these sub-populations all the time. Using a mixture model formulation, each sub-population is indexed by *i* = 1, 2, …, *N*, and described by three parameters (*μ*_*i*_, *v*_*i*_, *w*_*i*_): the mean *μ*_*i*_ and variance *v*_*i*_ of expression levels, and the relative frequency *w*_*i*_ of cells in the full population that belong to this sub-population. The latter is given by:

$$\begin{array}{c} w_{i}=\frac{n_{i}}{\sum_{j=1}^{N}n_{j}} \end{array}$$ (1)

where *n*_*i*_ is the number of cells in the *i*-th sub-population and $\sum_{j=1}^{N}n_{j}$ is the total number of cells in the full population. A related approach has been used by Gianola *et al*. [28] to study genetic parameters in the context of the quantitative genetics of mixture characters.

In the limit of large *N*, the parameters (*μ*_*i*_, *v*_*i*_, *w*_*i*_) describing a sub-population are taken as random variables (***μ***, ***v***, ***w***) (see Materials and methods for details of the notation used) following a particular multivariate distribution. Then, one can relate the mean *μ*_*F*_ and variance *v*_*F*_ of expression levels of the full population to the properties of the sub-populations, as detailed in S1 Text section A. Provided that there is no correlation between the frequencies (***w***) and either the means (***μ***), the squared means (***μ*^2^**) and the variances (***v***) of the sub-population, it follows that (S1 Text section A):

$$\begin{array}{cc} \mu_{F} & =E[x]=E[\mu ] \end{array}$$ (2)

$$v_{F}=V[x]=\underset{\begin{array}{c} \text{Within}\;\text{each}\\ \text{sub-population} \end{array}}{\underset{︸}{E[v]}}+\underset{\begin{array}{c} \text{Among}\\ \text{sub-populations} \end{array}}{\underset{︸}{V[\mu ]}}$$ (3)

where the subscript *F* is used to highlight that these are properties of the full population. Therefore, under these conditions, the mean of the full population is simply the expected value of the means of the sub-populations (E[μ]), while the variance of the full population is the sum of the variance within each sub-population (E[v]) and the variance among the sub-populations (variance in the means, V[μ]), thus taking the form of the general “law of total variance”.

Although Eqs 2 and 3 are general and independent of the precise definition of a sub-population, the two terms in Eq 3 suggest a specific definition, in which only the unstable component is present in each sub-population. In this way, the term of variation within any sub-population E[v] becomes the contribution of the unstable component to the variance of the full population, while the variation among the means of the sub-populations V[μ] is the contribution of the stable component. In the next section, expression levels within each sub-population will be described by a stochastic model, while the different sub-populations will have different means controlled by one of the parameters of this stochastic model.

#### An explicit model of protein expression in a cell population

**Variation within a sub-population**. The stochastic model of protein expression considered here is based on the work of Shahrezaei et al. [29], which has been followed by more recent studies (e.g. [30]). The model is defined by the following two equations:

$$\begin{array}{c} dx_{t}=\{\alpha \;\text{exp}(y_{t}-\frac{1}{2}\sigma^{2})-\frac{1}{\beta }x_{t}\}\;dt \end{array}$$ (4)

$$\begin{array}{c} dy_{t}=-\frac{1}{\tau }\;y_{t}\;dt+\frac{\sigma }{\sqrt{\tau /2}}dW_{t} \end{array}$$ (5)

where *x*_*t*_ is the amount of protein expressed at time *t*, and *y*_*t*_ is a stochastic variable following the Ornstein-Uhlenbeck process. In Eq 5, *W*_*t*_ is the Wiener process [31]. The parameters for the model are presented in Table 1, along with their respective dimensions.

**Table 1:** Description of the parameters of the stochastic model of protein expression defined by Eqs 4 and 5.

Parameter	Description	Dimensions
*α*	Mean protein production rate	Molecules/Time
*β*	Mean lifetime of the protein	Time
*σ*	Normalised dispersion of protein production rate	Non-dimensional
*τ*	Characteristic time of the fluctuations in protein production rate	Time

**Fig 1:**
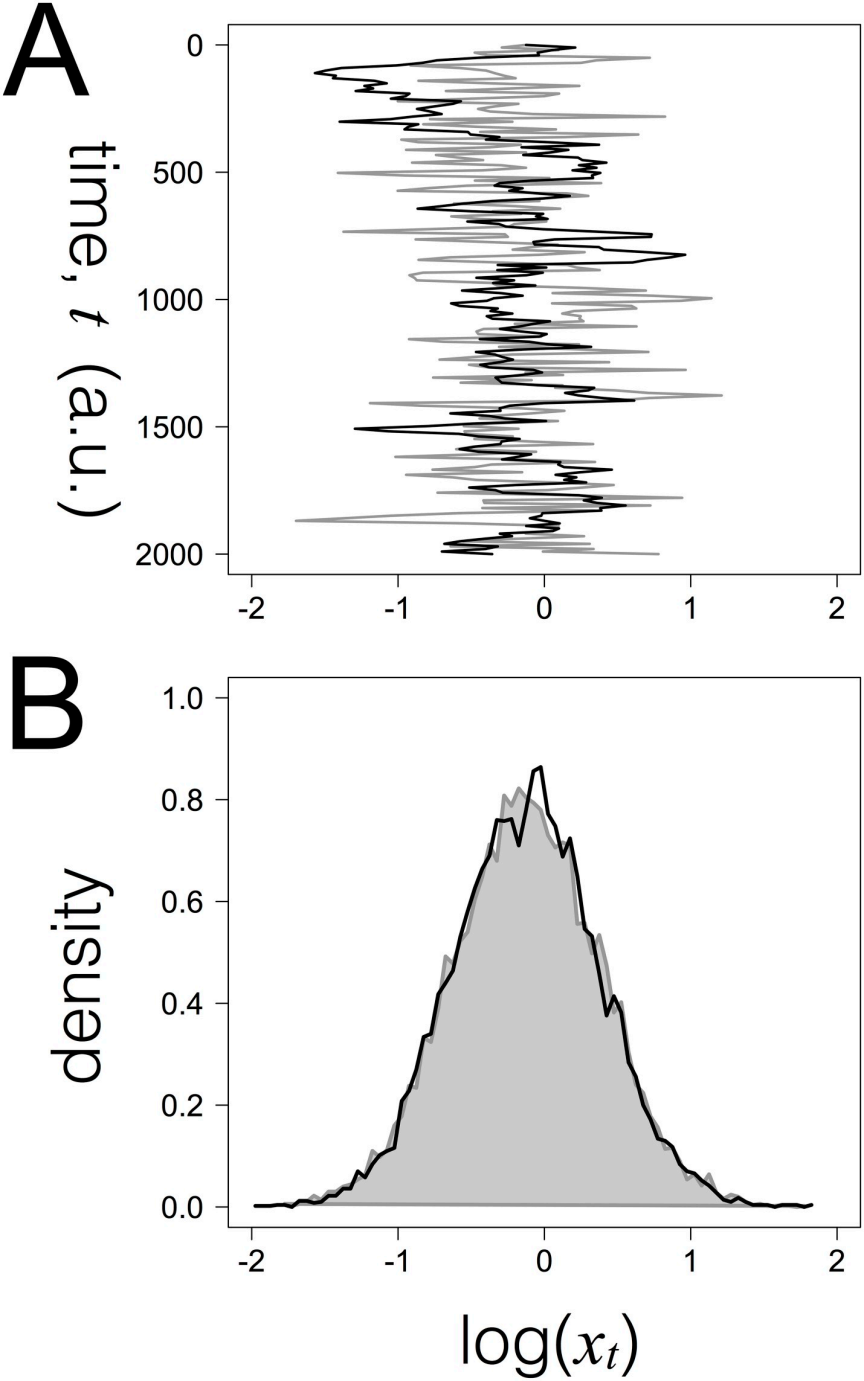
Dynamics of the protein expression levels *x*_*t*_ according to the stochastic model. A- Time courses of the log-transformed variable *x*_*t*_ obtained for two cells which differ in the characteristic time of the fluctuations (*τ* = 10 a.u. (grey) and *τ* = 100 a.u. (black)). The independent variable *t* is on the vertical axis and the log(*x*_*t*_) on the horizontal axis; B- Histograms of the log-transformed protein levels *x*_*t*_ in cell populations with slow and fast dynamics exemplified by the time courses. Each histogram is normalised by its maximum intensity and corresponds to 10000 independent realisations of the individual cell model sampled at time *t* = 200 a.u.; Remaining parameter values: *α* = 1., *β* = 1, and *σ* = 0.5.

The equation governing *dx*_*t*_ has two terms. The first term, $\alpha \exp(y_{t}-\frac{1}{2}\sigma^{2})$, is the rate of production which depends on the stochastic process *y*_*t*_, and the second term, *x*_*t*_/*β*, is the degradation rate following first-order kinetics with mean *β* protein lifetime. A model with a similar overall structure was reported before [32], in which mRNA transcription and degradation have also been explicitly incorporated. Eq 4 can be re-written as:

$$\begin{array}{c} \frac{dx_{t}}{dt}=\alpha \;z_{t}-\frac{1}{\beta }x_{t} \end{array}$$ (6)

where *z*_*t*_, defined as:

$$\begin{array}{c} z_{t}=\exp(y_{t}-\frac{1}{2}\sigma^{2}) \end{array}$$ (7)

denotes the instantaneous rate of protein production. This rate is normalised, to have unit expected value. All processes governing protein production (promoter transitions, transcription and translation, among others) are lumped together into the average rate *α* and the instantaneous rate given by *z*_*t*_. The representation in Eq 6, which highlights the contribution of lumped upstream factors, has been applied before in the analysis of models of stochastic gene expression (for example, [6, 7]). Eq 6 denotes that, in a single cell, the instantaneous rate of protein production is proportional to the instantaneous levels of these lumped upstream factors, and fluctuates as a function of time, with auto-correlation time approximately equal to *τ* [29]. These fluctuations are then propagated downstream, resulting in fluctuations in protein levels, with dynamics dictated by *τ* (through *z*_*t*_) and *β*. For simplicity, protein degradation is assumed to be deterministic, with the same rate 1/*β* for all cells. The temporal evolution of the protein expression levels *x*_*t*_ in two cells with distinct characteristic times *τ* is illustrated in Fig 1A.

It follows from Eq 7 that:

$$\begin{array}{c} z_{t}∼\mathrm{LN}(-\frac{1}{2}\sigma^{2},\sigma ),\;t\to \infty \end{array}$$ (8)

and therefore the stationary rate of protein production follows a lognormal distribution in cells of a sub-population, consistent with a report of lognormal rates of protein expression [33]. Eqs 4 and 5 are a simple model that generates, for a wide range of parameter values, a lognormal-like distribution of protein levels (Fig 1B), compatible with the widespread observation of the lognormal distribution in cell populations. In this scenario, in terms of the log-transformed protein levels (S1 Text section B), the mean and variance of a stationary sub-population are given by Eqs 9 and 10, respectively:

$$\begin{array}{c} \mu_{\text{log}}=E[\log(x_{t})]=\text{log}(\alpha \;\beta )-\frac{1}{2}\;\sigma_{W}^{2} \end{array}$$ (9)

$$\begin{array}{c} v_{\text{log}}=V[\log(x_{t})]=g\left(\sigma^{2},\tau /\beta \right)=\sigma_{W}^{2} \end{array}$$ (10)

where the subscript *W* will be used hereafter to denote that the variation is due to the stochastic process influencing the instantaneous rate of protein production. In Eq 10, *g*(⋅, ⋅) is an arbitrary function that can be estimated via simulation.

**Variation within and among sub-populations**. As formulated above, the stable component arises due to variation in the means of the sub-populations. Therefore, we assume that parameter *α* in Eq 4 is distributed in the full population, becoming a random variable, denoted by ***α***. Consequently, each sub-population is described by one value of *α*, resulting in different average rates of production, and hence different mean expression levels.

For simplicity, we consider the case that $\alpha ∼\mathrm{LN}(\mu_{\alpha },\sigma_{\alpha })$. For the *i*-th sub-population, with parameter *α*_*i*_, the mean and variance follow from Eqs 9 and 10:

$$\begin{array}{cc} \mu_{i,\text{log}} & =\log(\alpha_{i}\;\beta )-\frac{1}{2}\;\sigma_{W}^{2} \end{array}$$ (11)

$$\begin{array}{cc} v_{i,\text{log}} & =\sigma_{W}^{2} \end{array}$$ (12)

where $\sigma_{W}^{2}$ is assumed to be the same for all sub-populations. In terms of log-transformed values, plugging Eqs 9 and 10 into Eq 3, one obtains the variance of the full population:

$$\begin{array}{cc} v_{F,\text{log}} & =\sigma_{T}^{2}=\sigma_{W}^{2}+\sigma_{\alpha }^{2} \end{array}$$ (13)

An important property of Eq 13, which is based on log-transformed values, is that the parameters that represent the variances due to the stable and unstable components ($\sigma_{\alpha }^{2}$ and $\sigma_{W}^{2}$, respectively) remain separate. This is a key feature, greatly simplifying the process of analysis and inference throughout this work. As detailed in the S1 Text section C, the equivalent of Eq 13 considering protein levels without any transformation has an additional term, dependent on $\sigma_{\alpha }^{2}$ and $\sigma_{W}^{2}$. This additional term arises since the variance of each sub-population in this case depends on the value of *α*. Therefore, from this point on, we consider the analysis based on log-transformed values only.

### Isolating cells allows to quantify the contributions to the variation in a cell population

#### Definition of the relative contribution of the stable component

The variance of log-transformed expression levels of the full population is simply the sum of variances due to the stable and unstable components (Eq 13). In this context, in analogy with the *R*^2^ quantification of the variance explained by a linear regression model, we define $R_{\alpha }^{2}$ as:

$$\begin{array}{c} R_{\alpha }^{2}=\frac{\sigma_{\alpha }^{2}}{\sigma_{T}^{2}},\;0\leq R_{\alpha }^{2}\leq 1 \end{array}$$ (14)

to denote the proportion of the observed variance that is explained by the stable component.

Hence, $R_{\alpha }^{2}$ formalizes and quantifies the relative contribution of the stable component to the total variance of the full population, reducing the problem of quantifying the contributions to the estimation of a single parameter. In the case of $R_{\alpha }^{2}=0\%$, variation in expression levels arises exclusively due to the unstable component; conversely, the stable component explains all the observed variation if $R_{\alpha }^{2}=100\%$. Finally, in the intermediate case $0\%<R_{\alpha }^{2}<100\%$, a combination of the two components is at play.

#### The dynamics of the expression distribution of isolated cell cohorts depends on the relative contribution of the stable component

After defining $R_{\alpha }^{2}$, a setup for its estimation is derived. Since the original population is assumed to be a mixture of sub-populations, a natural approach for estimation is to isolate a cohort of cells and to follow the temporal evolution of some property of this cohort. The isolation of cells according to the expression levels of some protein has been described in previous experimental works [10, 14, 16–18], usually employing fluorescence-activated cell sorting (FACS). To simplify the presentation, it is assumed that the property is always quantified based on a sufficiently large number of cells, such that sampling effects are negligible.

Hereafter, a time reference *t* is defined beginning from the instant of isolation in a hypothetical experiment. Let an isolated cell cohort correspond to cells between percentiles *p*_1_ and *p*_2_ of expression levels of the original population. Without loss of generality, it is assumed hereafter that *p*_1_ < *p*_2_. Therefore, the two percentiles should satisfy 0% ≤ *p*_1_ < *p*_2_ < 100% or 0% < *p*_1_ < *p*_2_ ≤ 100%. This ensures that at least one of the isolated cohorts to be used for inference is not identical to the original population at time *t* = 0. Hence, isolating cells corresponds, indirectly, to selecting some of the sub-populations, if any compose the original population. Upon isolation, the expression levels of cells in a given sub-population will relax to the stationary distribution of that sub-population. Therefore, at the level of the isolated cohorts being tracked, changes in the property of expression levels are related to the dynamics of the unstable component, as expression levels of the sub-populations that have been isolated relax to their stationary values. The time for this relaxation to take place will be hereafter referred to as the characteristic time of the variation.

**Fig 2:**
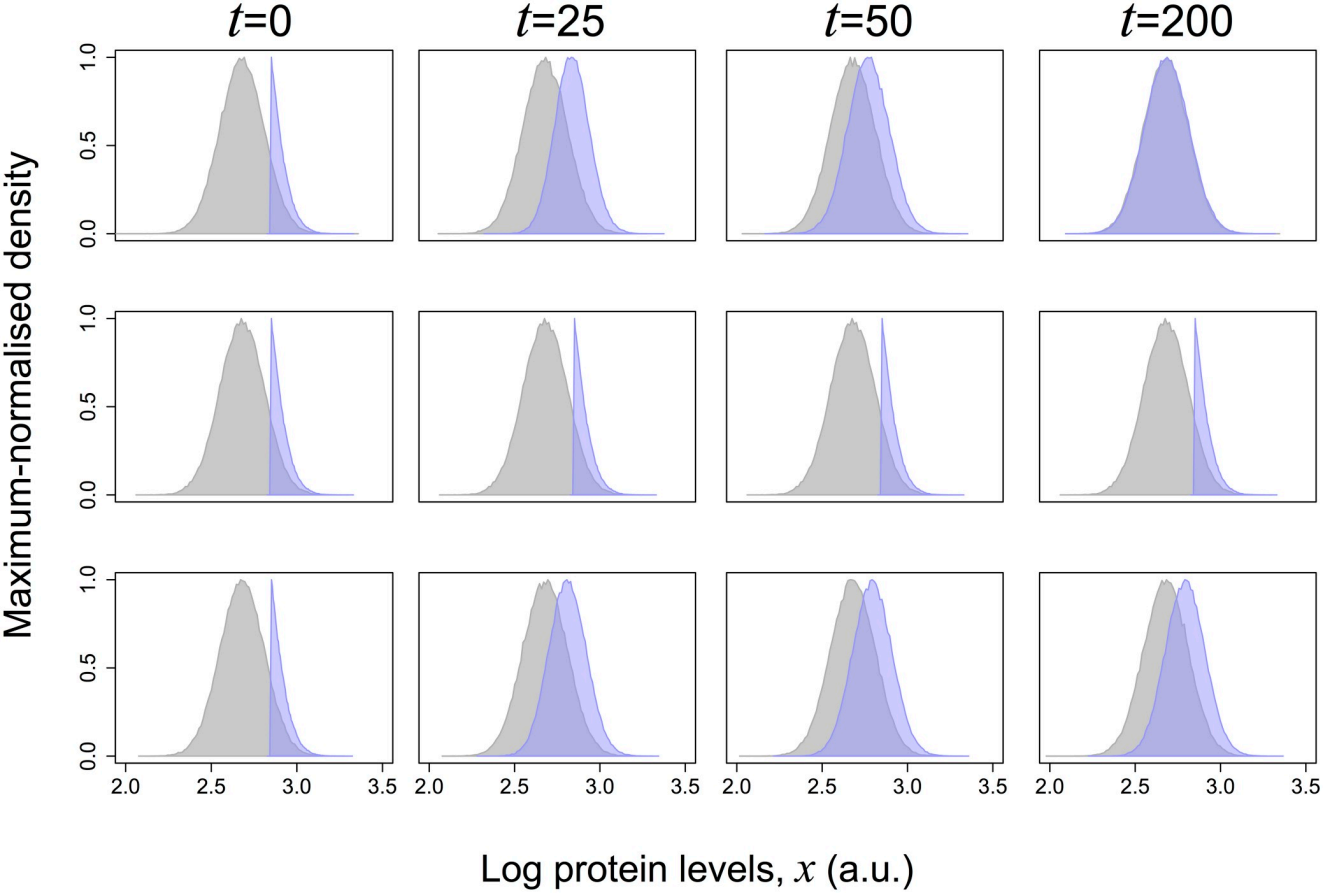
Simulation of the possible results obtained when a cohort of high expressor cells is isolated from a full population and followed in time. The graphs are histograms of the values of the expression levels variable *x* at the indicated times in 10000 independent realisations of the model for three values of $R_{\alpha }^{2}$ (0.0 (top), 1.0 (middle) and 0.25 (bottom)) simulating an isolated cohort of higher expressors (blue) or the full population from which the cohort was isolated (gray).

In a given experiment, three outcomes are possible (Fig 2). If only the unstable component is present ($R_{\alpha }^{2}=0$), after waiting a sufficiently long amount of time, the distribution of protein expression in the isolated cohort will converge to that of the original population (Fig 2, top). In contrast, if the observed variation is explained by the stable component only ($R_{\alpha }^{2}=100$), the distribution of the isolated cohort will not change as a function of time, remaining identical to that just after being isolated; it will always differ from that of the original population (Fig 2, middle). Finally, if both the stable and unstable components are present in the original population ($0<R_{\alpha }^{2}<100$), the isolated cohort will evolve in time, but without ever restoring the distribution of the original population (Fig 2, bottom).

The key question now is what properties of the isolated cohort can be used to infer $R_{\alpha }^{2}$. The next section shows that $R_{\alpha }^{2}$ can be accurately inferred from the dynamics of the means of the cohorts and examines the choice of a specific approach for isolation in term of the percentiles *p*_1_ and *p*_2_. The additional features that can be extracted from the variance of the isolated cohort are addressed in S1 Text section G.

### Estimating the relative contribution of the stable component

This section uses simulation to identify which property of the isolated cohorts can lead to a good estimate of $R_{\alpha }^{2}$, when followed in time. In the simulations, protein expression levels are described by the model derived above, neglecting cell division for simplicity. Since all derivations are based on Eq 13, the analysis herein relies on log-transformed values of protein levels.

The isolated cohort considered at first for inference here is composed of the 10% of cells with the highest (respectively lowest) expression levels in the original population hereafter referred to as “high expressors” (respectively “low expressors”). Following the notation introduced in the previous section, we have *p*_1_ = 90% and *p*_2_ = 100% (respectively, *p*_1_ = 0% and *p*_2_ = 10%). The choice of 10% is arbitrary, and is deemed to represent, at least in principle, a good compromise between resolution and number of cells obtained. Moreover, a random sample of the original population will serve as reference.

**Fig 3:**
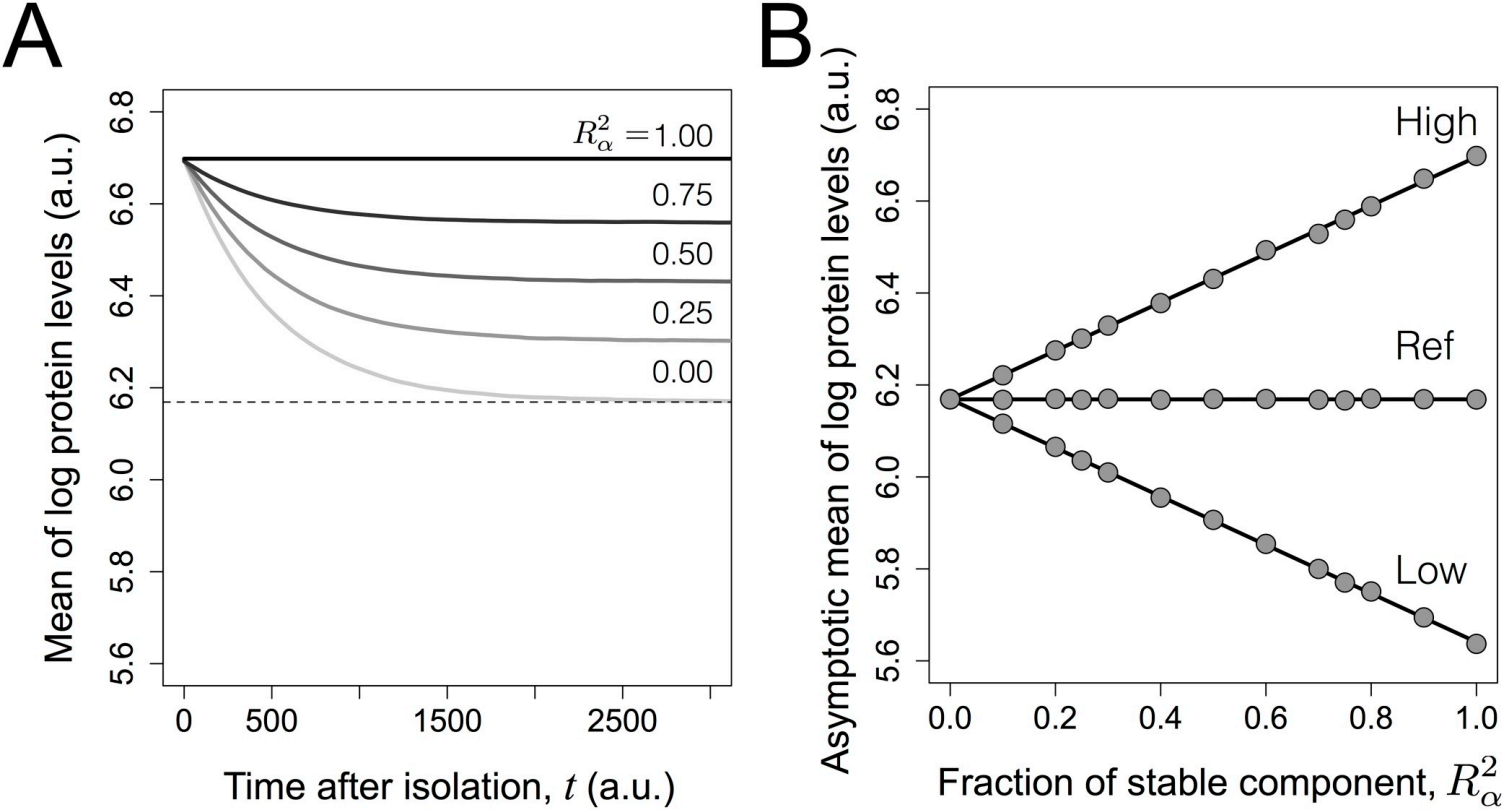
Simulation of transient dynamics and asymptotic limits of the mean protein expression in isolated cohorts. A- Dynamics of mean log protein expression levels of “high expressors” after isolation as 10% of original populations with different values of $R_{\alpha }^{2}$, but constant $\sigma_{T}^{2}$; B- Asymptotic mean of 10% low expressors, 10% high expressors and reference population as a function of $R_{\alpha }^{2}$ in the simulations. The symbols represent simulation results, while the lines represent the best-fit of a straight line. Error bars are not represented for simplicity. Remaining parameter values: *τ* = 500, *β* = 5 and *σ*_*T*_ = 0.3.

We first address how the dynamics of the mean of log-transformed protein levels in isolated cohorts, shown in Fig 3A for the high expressors. Briefly, the mean of an isolated cohort will evolve smoothly until it reaches an asymptotic limit.

It turns out that the asymptotic mean of log protein levels of an isolated cohort is a linear function of the $R_{\alpha }^{2}$ of the original population from which it was obtained, as illustrated in Fig 3B in the cases of the isolation of the 10% high and low expressors in populations with different $R_{\alpha }^{2}$. This linear relationship allows one to define a straightforward approach for estimating $R_{\alpha }^{2}$. Defining Δ_*A*,*B*_(*t*) as the difference between the means of log-transformed values of two isolated cohort *A* and *B*, respectively *μ*_*A*_(*t*) and *μ*_*B*_(*t*), at time instant *t*:

$$\begin{array}{c} \Delta_{A,B}\left(t\right)=\mu_{A}\left(t\right)-\mu_{B}\left(t\right) \end{array}$$ (15)

then, $R_{\alpha }^{2}$ can be estimated via:

$$\begin{array}{c} R_{\alpha }^{2}=\frac{\lim_{t\to \infty }\Delta_{A,B}\left(t\right)}{\Delta_{A,B}\left(0\right)},\;\Delta_{A,B}\left(0\right)\neq 0 \end{array}$$ (16)

as demonstrated analytically in S1 Text section D.

The condition Δ_*A*,*B*_(0) ≠ 0 for using Eq 16 implies that the two isolated cohorts being compared must have different means just after isolation (*t* = 0). From the inequality in Eq 14, an additional relationship for Δ_*A*,*B*_(*t*) holds:

$$\begin{array}{c} \lim_{t\to \infty }(\Delta_{A,B}\left(t\right))\leq \Delta_{A,B}\left(0\right) \end{array}$$ (17)

Therefore, the stationary difference between the means of log-transformed expression levels of the isolated cohorts *A* and *B* is expected to be, under the present formulation, lower than or equal to the difference immediately after isolation. Therefore, a key result is that, to estimate $R_{\alpha }^{2}$, one may simply calculate the ratio between the asymptotic value of difference between the means of log-transformed protein levels in two isolated cohorts relative to its initial value after isolation.

An important consequence for experimental design is that one can improve the resolution in the estimation of $R_{\alpha }^{2}$ by maximising the value of Δ_*A*,*B*_(0). For any given percentage of cells to be isolated (chosen as *p*_2_ − *p*_1_), the maximal initial difference is obtained by isolating the extreme high and low expressors. Consequently, the remainder of this work focuses on this case, by always relying on the function Δ_*H*,*L*_(*t*) for estimation, where H and L denoted respectively the high and low expressors.

Eq 16 has an important advantage from an experimental point of view: the fact that it depends only on the differences between the means of the sorted and reference populations. This is particularly important given that there are typically day-to-day systematic variations in the absolute values read by a flow cytometer, to which Eq 16 is robust. On a similar vain, by relying on means the analysis is robust to the random measurement errors of the flow cytometers. However, it is essential that measurements used to calculate and analyse the cohorts means are independent of the measurements used for sorting such that the respective measurement errors are uncorrelated; otherwise, the value of Δ_*H*,*L*_(0) will be offset to higher values by sampling the tails of sorting measurement errors, leading to underestimates of $R_{\alpha }^{2}$ due to the statistical effect of regression to the mean.

The asymptotic analysis just presented does not allow to consider the dynamics of the expression levels. To address these dynamics we introduce the time-dependent function Ω_*H*,*L*_(*t*) given by:

$$\begin{array}{c} \Omega_{H,L}\left(t\right)=\frac{\Delta_{H,L}\left(t\right)}{\Delta_{H,L}\left(0\right)},\;\Delta_{H,L}\left(0\right)\neq 0 \end{array}$$ (18)

**Fig 4:**
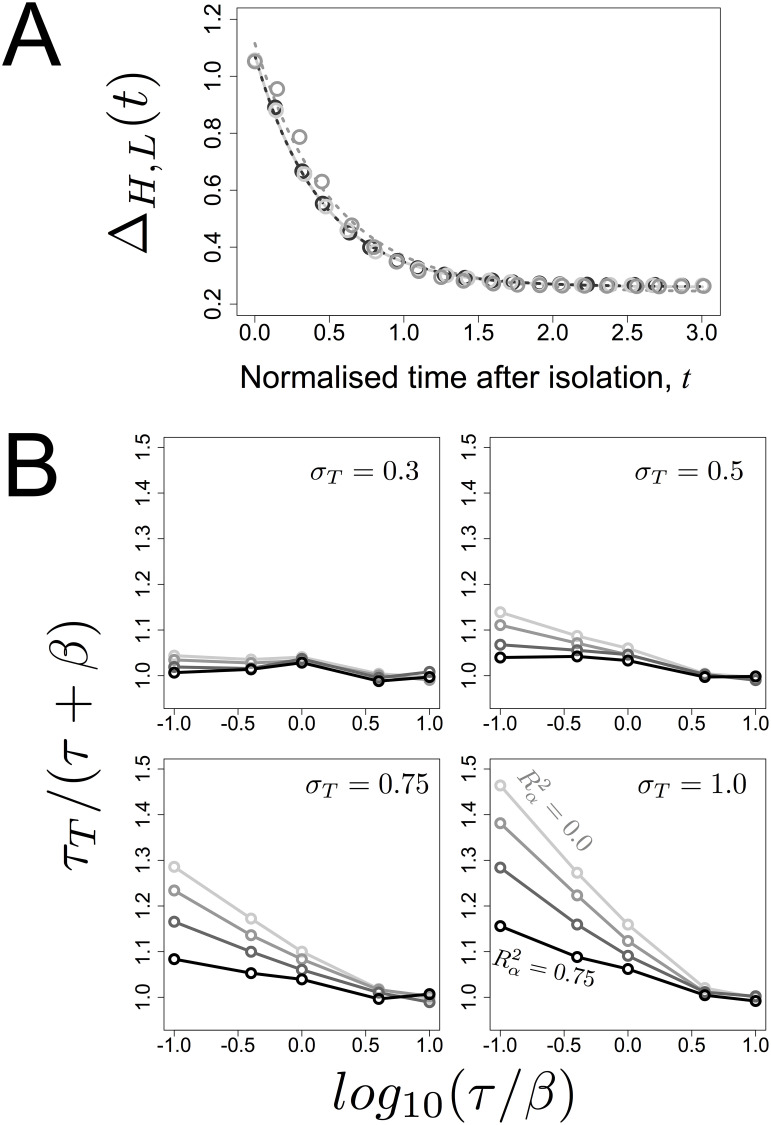
The function Δ_*H*,*L*_(*t*) decays with approximately exponential dynamics. A- Simulations of the isolation of cells were done, for various values of *τ* and *β*, with $R_{\alpha }^{2}=25\%$. Shown are simulation results (symbols), along with the results of fitting the model of exponential decay Δ(*t*) = *a* + *b* exp(−*t*/*τ*_*T*_) to the simulation data (dashed lines), where *a* and *b* are constants. Time is normalized in each case by the instant t* such that Δ_*H*,*L*_(t*) has decayed by 90%. The light to dark gray tones correspond to the values of the ratio *τ*/*β* = 0.1, 1.0, 10.0 respectively, with *β* = 50; B- Comparison between *τ* + *β* and the value estimated for *τ*_*T*_. Simulated data (Δ_*H*,*L*_(*t*)) were fitted under the same setup as in (A) and the resulting values of *τ*_*T*_ plotted as a function of the value of *τ* + *β*. Each graph corresponds to simulations using the indicated value of *σ*_*t*_ with different values of $R_{\alpha }^{2}$ (0.0, 0.25, 0.50 and 0.75) depicted in different gray tones (the darker the tone the higher the value of $R_{\alpha }^{2}$).

Being based on the means of log-transformed values of two populations that have been isolated, Δ_*H*,*L*_(*t*) follows an approximately exponential decay (Fig 4; see section F in S1 Text for a rationale). Using the approximation of exponential decay, and defining the effective characteristic time as *τ*_*T*_, Ω_*H*,*L*_(*t*) takes the following form:

$$\begin{array}{c} \Omega_{H,L}(t)=\frac{\Delta_{H,L}(t)}{\Delta_{H,L}(0)}=\underset{\begin{array}{c} \text{Relative}\\ \text{contribution}\\ \text{of the stable}\\ \text{component}\\ \text{in the original}\\ \text{population} \end{array}}{\underset{︸}{R_{\alpha }^{2}}}+\underset{\begin{array}{c} \text{Relative}\\ \text{contribution}\\ \text{of the unstable}\\ \text{component}\\ \text{in the original}\\ \text{population} \end{array}}{\underset{︸}{\left(1-R_{\alpha }^{2}\right)}}\;\underset{\begin{array}{c} \text{Relaxation}\\ \text{of the unstable}\\ \text{component}\\ \text{(timescale}\\ \text{term)} \end{array}}{\underset{︸}{\exp(-t/\tau_{T})}} \end{array}$$ (19)

It follows that the effective characteristic time *τ*_*T*_ is undefined in the case of $R_{\alpha }^{2}=100\%$, since Δ_*H*,*L*_(*t*) does not change as a function of time after isolation. Since *τ*_*T*_ is a measure of the time needed for the initial difference Δ_*H*,*L*_(0) to reach the asymptotic value lim_*t*→∞_ {Δ_*H*,*L*_(*t*)}, it provides a formal characterisation of the timescale of the variation.

An exhaustive simulation study (Fig 4B) led to the conclusion that *τ*_*T*_ can be approximated, with a typical bias of at most 5–10% of the true value, as:

$$\begin{array}{c} \tau_{T}\approx \beta +\tau \end{array}$$ (20)

Therefore, the auto-correlation time of the stochastic rate of protein production (*τ*) and the mean lifetime of the protein (*β*) determine the timescale of the variation in expression levels (*τ*_*T*_).

**Fig 5:**
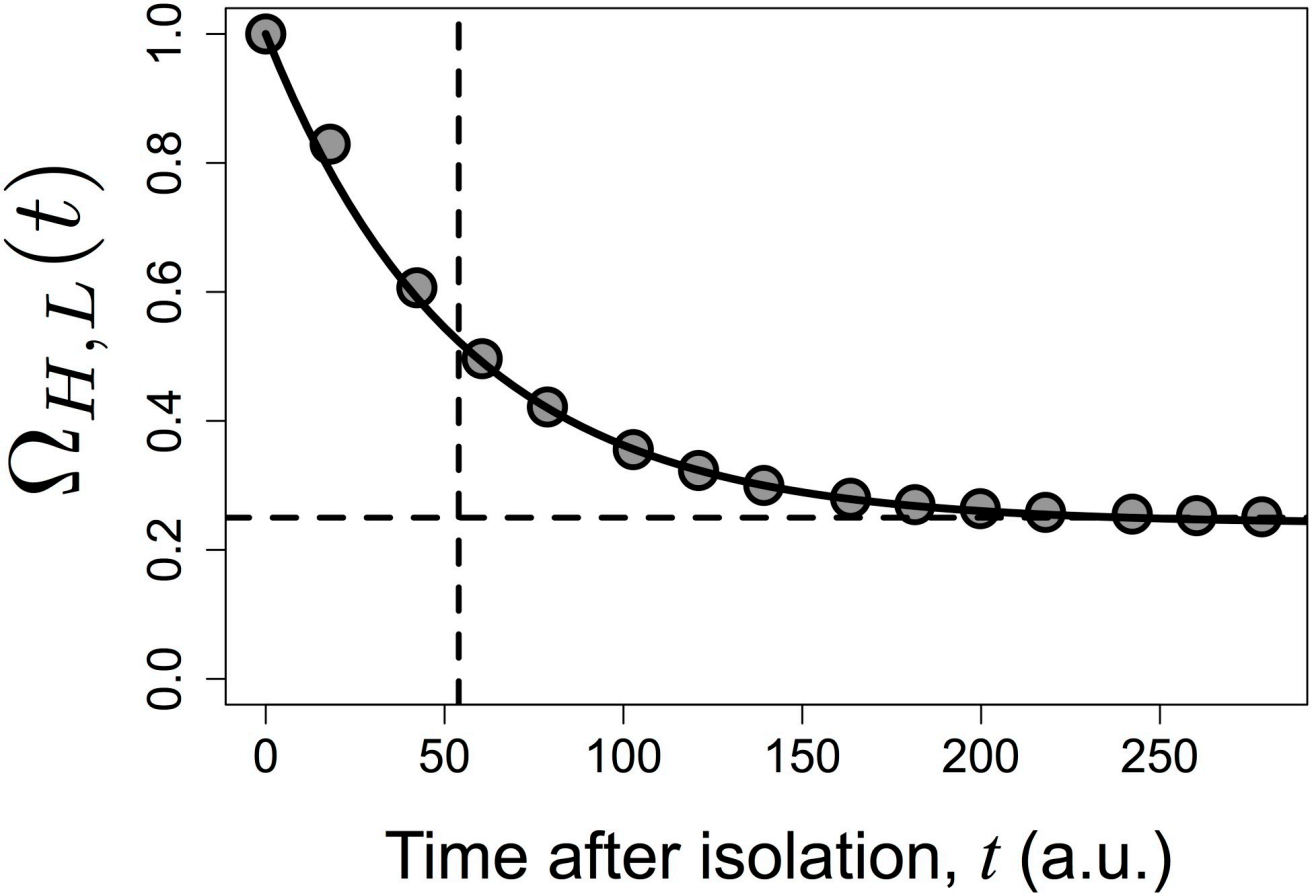
Illustration of function Ω_*H*,*L*_(*t*). Shown are simulation results (symbols), with $R_{\alpha }^{2}=25\%$, *τ* = 50 and *β* = 5, which were fitted to the expression for Ω_*H*,*L*_(*t*) in Eq 19 (continuous line). The horizontal and vertical dashed lines indicate respectively the true value of $R_{\alpha }^{2}$ and the value of *τ*_*T*_, as given by Eq 21.

The relative contribution of the stable component ($R_{\alpha }^{2}$) and the effective characteristic of the variation (*τ*_*T*_) can be visualized in a single plot, derived from Eq 19. As shown in Fig 5, $R_{\alpha }^{2}$ corresponds to the asymptotic value of Ω_*H*,*L*_(*t*), while *τ*_*T*_ corresponds to the instant of time that satisfies:

$$\begin{array}{c} 1-\Omega_{H,L}\left(\tau_{T}\right)=(1-\exp(-1))\;(1-R_{\alpha }^{2})\approx 0.63\;(1-R_{\alpha }^{2}) \end{array}$$ (21)

Since Eq 19 features an exponential decay, it follows that the plateau is reached in practice after an amount of time approximately equal to 5*τ*_*T*_. Furthermore, the inequality in Eq 17 becomes:

$$\begin{array}{c} \Delta_{H,L}\left(t\right)\leq \Delta_{H,L}\left(0\right)\;\forall t \end{array}$$ (22)

since function Δ_*H*,*L*_(*t*) is monotonically decreasing with time.

Although this section has focused on the case in which high and low expressors are used, all the properties derived also hold for any two isolated cohorts *A* and *B*. The only requirement is that the condition Δ_*A*,*B*_(0) ≠ 0 is satisfied.

### Quantification of the components shaping the variation in T-cell receptor expression levels

The theoretical framework developed in the previous sections is used here in the analysis of the variation in the expression of the TCR in mouse CD4+ T lymphocytes. The TCR is a heterodimeric membrane receptor that elicits signal transduction upon interaction with MHC-peptide complexes on the membrane of antigen-presenting cells [34]. In wild-type animals, the T cell populations are genetically heterogeneous at the TCR level, due to the somatic recombination at the loci encoding the receptor chains in thymocytes (reviewed in [35]). In contrast, genetically manipulated mouse strains are available in which all the T cells express the same TCR (for example, [27]). In these mouse strains, the somatic recombination is ablated (*Rag2^−/−^* background) and a single functional TCR is expressed in all cells driven by transgenes encoding the two chains of the TCR.

**Fig 6:**
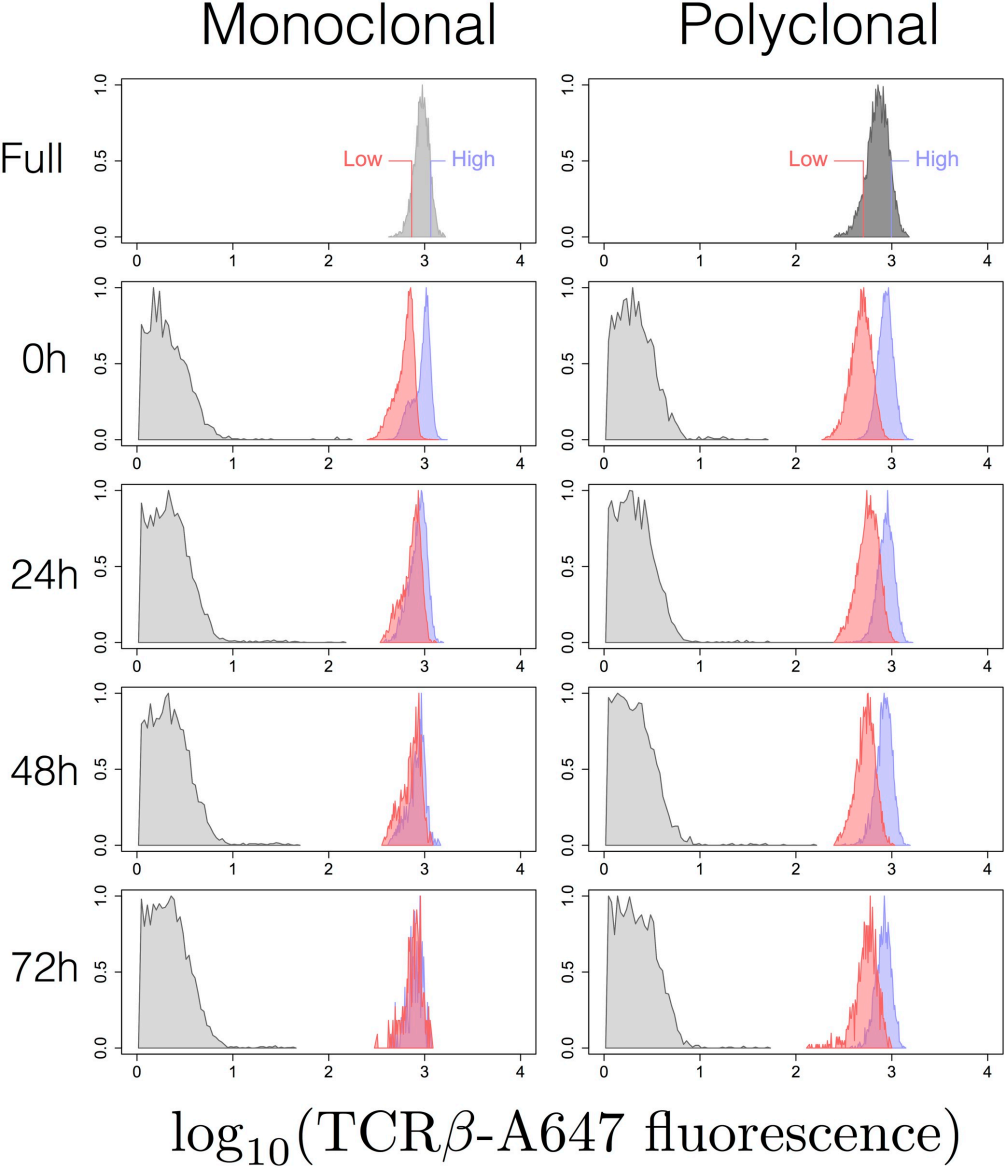
Dynamics of TCR expression in high and low expressor cohorts sorted from monoclonal (Marilyn; left) and polyclonal (wildtype; right) populations. The graphs are the histograms of frequency of log-transformed TCR fluorescence in the high (blue) and low (red) expressors measured by flow cytometry at the indicated times after sorting. Unstained population is also shown in gray.

To quantify the origin and timescale of the variation in the context of the TCR, we used a polyclonal population from a wild-type inbred strain and a surrogate monoclonal population from the Marilyn TCR-transgenic strain [27]. In this setup, we are interested in comparing the values of $R_{\alpha }^{2}$ and *τ*_*T*_ estimated for the polyclonal and the Marilyn monoclonal populations. These two populations show comparable mean expression values (see Fig 6, top) but the expression is more variable in the polyclonal population than in the monoclonal one [36], presumably reflecting the genetic diversity [37].

In theoretical framework, the stable component arises from different mean protein production rates. In polyclonal populations, the stable variation in average TCR production may be caused by the differential regulation of expression of the receptor sub-units, depending on the specificity of the particular TCR, or by the differential ability of the specific sub-units to pair and be expressed [38]. In any case, genetic heterogeneity would ultimately explain some of the variation observed at the level of a polyclonal population. If so, this would imply that $R_{\alpha }^{2}>0$ for a polyclonal population. By analysing a TCR-transgenic population, we addressed whether genetic variation is the only factor explaining the stable component. In the affirmative case, one would obtain $R_{\alpha }^{2}=0$ for a TCR-transgenic population. If one obtains $R_{\alpha }^{2}>0$, non-genetic mechanisms must be evoked.

We adopted an experimental design in which high and low expressors, defined to contain 10% of the mass of the starting population distribution, were sorted (Fig 6, top) and then maintained *in vitro* without any stimulation. As described before [39], there was no cell division under these conditions, and cells slowly died off, such that after 3 to 4 days no live cells were left (the increased jaggedness of the histograms in Fig 6 reflects the decreased number of viable cells with time). Since in the Marilyn transgenic strain, all T cells have a naive phenotype [27], we restricted the analysis of the wildtype polyclonal populations to those cells that express high levels of the CD45RB marker, indicative of a naive phenotype [40]. By restricting the analysis to naive cells, the distribution of cell size as measured by Forward Scatter was similar in the high and low expressor cohorts when sorted from the monoclonal Marilyn population as well as from polyclonal population (S1 Text section H).

**Fig 7:**
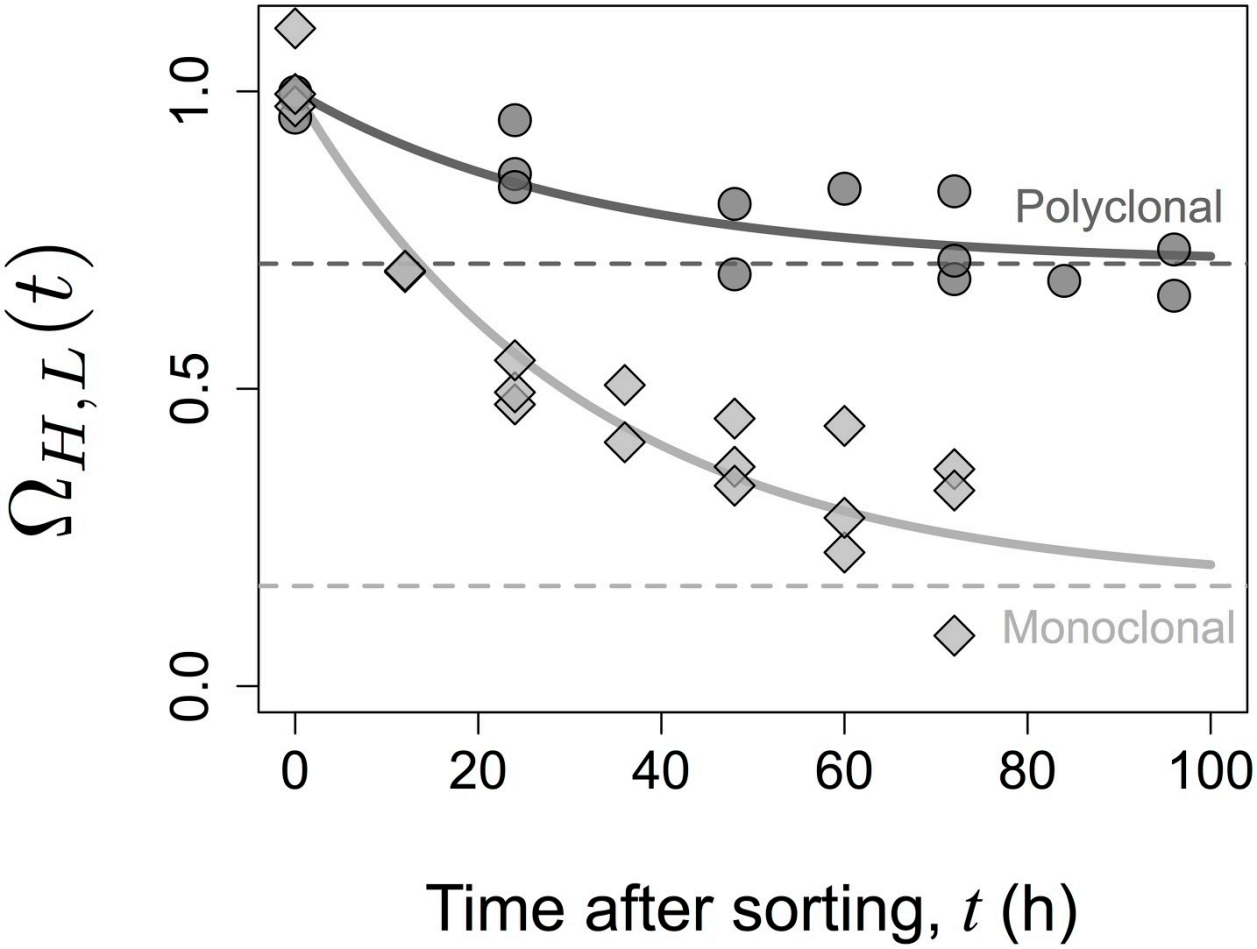
Dynamics of Ω_*H*,*L*_(*t*) in sorted cohorts. The symbols are the point estimates of Ω_*H*,*L*_(*t*) = Δ_*H*,*L*_(*t*)/Δ_*H*,*L*_(0) at different times after sorting for polyclonal (circles) and monoclonal (diamonds) cell population data sets. The curves represent the best fit of the function Ω_*H*,*L*_(*t*), as defined by Modelling Scenario 2, to the ensemble of the populations data sets. The horizontal dashed lines indicate the estimates of the asymptotic $R_{\alpha }^{2}$.

**Fig 8:**
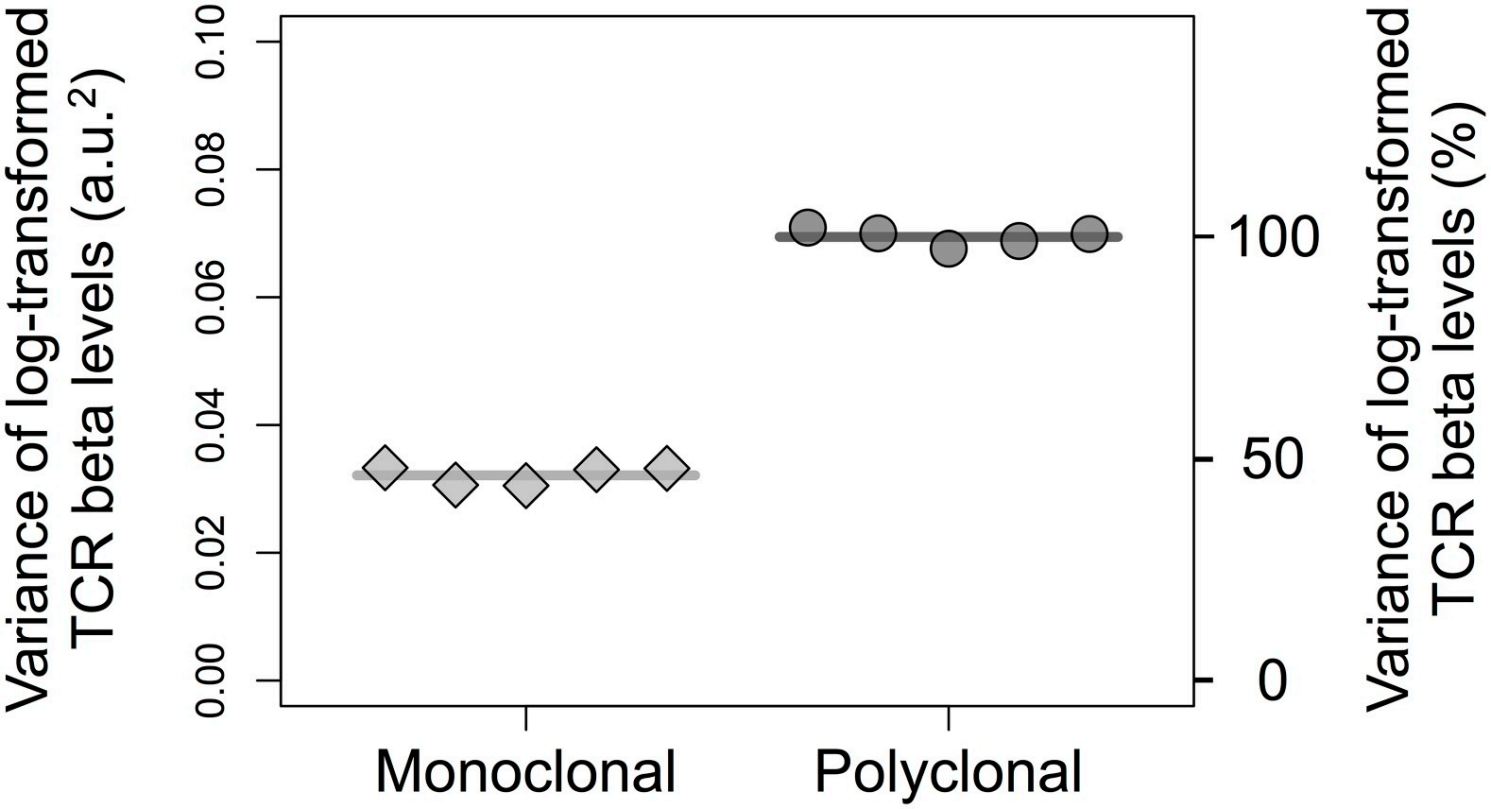
Variance of the log-transformed TCR expression levels in monoclonal (Marilyn; diamonds) and polyclonal (wildtype; circles) CD4+ T lymphocyte populations. The points are estimates of the variance in independent samples and the lines are the average value of these variances.

The dynamics of the frequency distribution of the TCR expression levels in cohorts of high and low expressors sorted from polyclonal and monoclonal animals and subsequently cultured *in vitro* for up to 72h is illustrated in Fig 6 for one of three independent experiments (represented in Fig 7). The distributions of the TCR expression levels in the high and low expressors sorted from wildtype polyclonal population remain clearly different. In contrast, the high and low expressors from the monoclonal Marilyn TCR-transgenic population become very similar as a function of time after sorting. The values of Ω_*H*,*L*_(*t*) in the three experimental data sets are shown in Fig 7. It is worth noticing that the Marilyn histogram data set exemplified (Fig 6) happens to show the highest convergence of the high and low expressors distributions at the last time sampled. Also, the normalisation (Eq 18) masks the fact that the value of Δ_*H*,*L*_(0) was conspicuously greater for the polyclonal population in accordance with the observations that the variance ($\sigma_{T}^{2}$) is larger in polyclonal populations than in TCR transgenic populations (Fig 8; and also [36]).

To estimate $R_{\alpha }^{2}$ and *τ*_*T*_ by fitting the model to the experimental data Eq 19 must be refined as follows:

$$\begin{array}{c} \Delta_{H,L}\left(t\right)=\delta_{0}(R_{\alpha }^{2}+(1-R_{\alpha }^{2})\;\exp(-t/\tau_{T})) \end{array}$$ (23)

where *δ*_0_ represents an estimate, obtained via fitting, of the “true” initial value Δ_*H*,*L*_(0). Eq 23 has the important property of preserving the statistical independence between data points used as input for the fitting, a key requirement for proper statistical analyses.

The analysis was based on fitting the three-parameter exponential model (Eq 23) to the ensemble of the data, composed of the multiple experiments done for each biological population. The different modelling scenarios being tested are defined by specifying each of the three parameters, $R_{\alpha }^{2}$, *τ*_*T*_ and *δ*_0_, for each biological population as being shared or not between the polyclonal and monoclonal populations. Small variations in defining the percentages for sorting high and low expressors in different experiments are expected to sporadically affect the value of *δ*_0_ and therefore this parameter was always fitted separately for each experiment. The modelling scenarios are obtained by specifying how parameters $R_{\alpha }^{2}$ and *τ*_*T*_ are shared between the biological populations. The complete description of the modelling scenarios considered is presented in Table 2 (column 2). Modelling Scenario 1 represents the null model, according to which the polyclonal and monoclonal populations are described by the same values of $R_{\alpha }^{2}$ and *τ*_*T*_. This is the scenario with the smallest number of parameters considered. Scenario 2 represents the plausible situation in which these two populations may be described by different values of $R_{\alpha }^{2}$, but equal *τ*_*T*_, while in Scenario 3 parameter *τ*_*T*_ is also allowed to be different in the two populations. Finally, Scenario 4 represents a lower bound in terms of the error in the fitting, where data from each experiment is fitted independently, and has the largest number of parameters. The Akaike Information Criterion (AIC) [41] is used to compare the different modelling scenarios in their capacity to fit to the ensemble of the data. The AIC has a solid foundation on information theory [41], representing a compromise between the error in fitting the data and the number of parameters in the model. The results are presented in terms of the difference ΔAIC_*c*_ between the AIC for each Scenario and that of Scenario 1. In comparing different modelling scenarios, the one with the smallest value of the AIC (and therefore, the smallest value of ΔAIC_*c*_) provides the best and most parsimonious description of the data.

**Table 2:** Overview of the Modelling Scenarios tested, with a description of how parameters $R_{\alpha }^{2}$ and *τ*_*T*_ were set in the two biological populations, and the resulting number of parameters that are fitted. As discussed in the text, parameter *δ*_0_ were fitted separately for each experiment.

Scenario	Description	# Parameters Fitted
1	$R_{\alpha }^{2}$ and *τ*_*T*_ have the same values in the two biological populations	8
2	$R_{\alpha }^{2}$ may have different values for each biological population, but *τ*_*T*_ has the same value	9
3	Both $R_{\alpha }^{2}$ and *τ*_*T*_ may have different values for each biological population	10
4	Each experiment was fitted independently	18

The results of the model fitting, including the sum of squared residuals, point estimates of the parameters, and the value of ΔAIC_*c*_, are shown for each scenario in Table 3. It follows that Scenarios 2 and 3 have the lowest values of ΔAIC_*c*_, with Scenario 3 having a slightly higher value. Since the latter has one extra parameter, this suggests that a scenario where only $R_{\alpha }^{2}$ is allowed to be different constitutes the most parsimonious explanation for the data. Hence, altogether Scenario 2 is favoured, according to which the two populations differ only in $R_{\alpha }^{2}$. In this case, we obtain an effective timescale of 32 hours, and values of $R_{\alpha }^{2}$ of 71% for the polyclonal and 17% for the monoclonal population, with 95% confidence intervals of [57%, 79%] and [0%, 31%], respectively. Finally, the function Ω_*H*,*L*_(*t*) resulting from scenario 2 is shown in Fig 7, highlighting the values of $R_{\alpha }^{2}$ estimated for each population.

**Table 3:** Estimates for the parameters of the populations obtained by fitting the data on Δ_*H*,*L*_(*t*), based on the different modelling scenarios under consideration. The results are presented in terms of ΔAIC_*c*_, the difference between the value of the AIC (corrected for small sample size; see Materials and methods) of each scenario and scenario 1. Modelling scenarios with lower values of ΔAIC_*c*_ provide a more parsimonious explanation for the data.

Fitting Δ_*H*,*L*_(*t*)							
Scenario	SS Residuals	Population	Exp. #	*δ*_0_	$R_{\alpha }^{2}$ (%)	*τ*_*T*_ (h)	ΔAIC_*c*_
1	0.103	Polyclonal	1	0.55	61	32	0.0
			2	0.62			
			3	0.63			
		Marilyn	1	0.24			
			2	0.29			
			3	0.26			
2	0.028	Polyclonal	1	0.52	71	32	-40.5
			2	0.58			
			3	0.59			
		Marilyn	1	0.37	17		
			2	0.40			
			3	0.37			
3	0.025	Polyclonal	1	0.51	59	75	-39.6
			2	0.57			
			3	0.58			
		Marilyn	1	0.37	24	24	
			2	0.41			
			3	0.38			
4	0.015	Polyclonal	1	0.50	22	199	2.2
			2	0.57	67	46	
			3	0.57	0	249	
		Marilyn	1	0.40	4	32	
			2	0.39	40	16	
			3	0.37	25	27	

## Discussion

In this article, we introduce a new approach to analyse the variation in protein expression levels in a cell population, which enables measuring the characteristic dynamics of the fluctuations in cellular expression and estimating the magnitude of stable and unstable contributions to the variation across cells. The analysis is based on the realisation that the difference between the means of log-transformed expression levels in two selected cohorts isolated from a population of interest converges with approximate exponential dynamics to an asymptotic value. By normalising this asymptotic value by the difference in cohorts’ means immediately after their isolation one obtains an unbiased estimation of the proportion of population variance that is explained by the stable component $R_{\alpha }^{2}$, while the mean convergence time *τ*_*T*_ measures the timescale of unstable component dynamics. This key insight stems from perceiving any cell population as a mixture of many independent subpopulations, each with a characteristic mean expression level, that is fixed yet distributed among the subpopulations. Under these assumptions, the population variance is equated to the sum of the variance of the subpopulations means, which embodies the stable component of variation, and the variance of the expression level within the subpopulations, which represents the unstable component.

At first sight, the stable and unstable components of expression variation, as formulated here, are analogous to what Huang [10] referred to as population and temporal noise, respectively. However, this analogy is not straightforward. Huang’s definition of population noise precludes, by construction, any underlying genetic and stable epigenetic mechanisms. In contrast, the stable component, as defined here, is a statement about the dynamics of variation and is silent about mechanism. We believe that the terms stable and unstable components are not only intuitive but convey a more precise description of variation in terms of its temporal dynamics. The mechanistic bases of these components remain a matter for further analysis. Putative mechanisms underlying the stable component include genetic variation and non-volatile epigenetic traits [10, 42]. In turn, the unstable component may be explained by noise in gene expression [9, 10], transient metastable epigenetic variants [10, 42] or noise in the partitioning of cellular contents during cytokinesis [43]. Stable gene expression variants, which would be part of stable component of variation, are expected to be pervasive, since differentiation stages, cell lineages and cell types are hallmarks of multicellular organisms [25, 26]. In spite of this expectation, most quantitative approaches to expression variation in cells in the past have focused on noise in gene expression [4, 7, 19, 21, 23, 24].

Measuring the extent to which selected cohorts of cells can restore the full complexity of the population from which they were sorted is an intuitive approach to analyse the heterogeneity of a population. This basic intuition motivated the experimental design used in several reports [14, 16–18, 44], in which the stable and unstable components of variation were evoked and utilised in an informal way. The capacity of cohorts to restore totally or partially the distribution of the original population has often been interpreted and discussed qualitatively, based on the visual inspection of raw flow cytometry histograms or of summary data time series. The present report advanced beyond such “half-full / half-empty glass” interpretations of data by contributing a rigorous quantitative method to analyse these kind of sorted cohort experiments based on the estimation of the two parameters, $R_{\alpha }^{2}$ and *τ*_*T*_, that encapsulate respectively the heterogeneity and the dynamics of the expression variation.

The method has limitations and constraints since it was tailored to the analysis of long-tailed unimodal expression distributions characteristic of most constitutive proteins, such as the T cell receptor. Applying this method requires conforming to the model assumptions and also some caution with the sampling procedures, like any other inference method. The question of how relaxing the assumptions affects the theoretical results, such as the convergence of Ω_*H*,*L*_(*t*) to $R_{\alpha }^{2}$, as well as the accuracy of estimates, deserves a systematic analysis that is beyond our present scope. A preliminary simulation analysis indicates that the equality $\Omega_{H,L}\left(\infty \right)=R_{\alpha }^{2}$ may be fairly robust to mild violations of model assumptions. This asymptotic equality seems to hold if one assumes a narrow distributed sub-population variance ($\sigma_{W}^{2}$), correlated or uncorrelated with the sub-population mean, as well as if one takes a subpopulation means distribution with a different shape, provided that the full distribution is approximately normal in logarithmic scale (S1 Text section E). The method may be repurposed to deal with multimodal expression distributions and multiple time scales (e.g. stable, slow and rapid variation dynamics) by modelling the full population as hierarchical mixture of sub-populations and allowing cells to flow between sub-populations. The additional complexity will demand specifying non-trivial assumptions about the structure of the cell flow function. At present, the population model assumes that the sub-populations are independent and at fixed density, making it especially suited to analyse the expression variation in the abundant quiescent cell populations and tissues of multicellular organisms. The values of $R_{\alpha }^{2}$ and *τ*_*T*_ estimated in proliferating cells will reflect not only cell-intrinsic stable and unstable variation but also differential fitness effects and noise in cell content partitioning upon cytokinesis [43]. The aggregate contribution of these confounding factors can be empirically quantified by comparing the $R_{\alpha }^{2}$ and *τ*_*T*_ values for the same cell types under proliferating and quiescent conditions.

The analysis method is grounded on a stochastic modelling framework. The protein expression levels in a single cell are described as very simple stochastic processes, based on [29], in which the instantaneous protein production rate (captured by variable *z*_*t*_) fluctuates generating a stationary log-normal distribution of expression levels in each subpopulation. Protein expression has been modelled by others considering transcriptional burst dynamics that can be shown to generate discrete numbers of transcripts following a negative binomial distribution. Although transcript copy number distributions are generally assumed to be described by a negative binomial distribution at any range of expression levels, they are well-approximated by a log-normal distribution at high copy numbers per cell [45]. Therefore the log-normal approximation underlying the dynamics of *z*_*t*_ is justified by the observation that the transcripts encoding the TCR *α* and *β* chains are among the most abundant in the cell [46]. This model of the single cell expression dynamics was used to simulate a population formalised as a large mixture of independent subpopulations. By applying the equation for partitioning the variance to this mixture model, the analysis based on log-transformed values emerged as the best approach, since in this case the contributions due to the stable and unstable components are additive, greatly simplifying inference. This is particularly relevant for flow cytometry data, which is typically analysed in a logarithmic scale. It is interesting to note that [18] also relied on log-transformed values for quantification, based on the analysis of time-series of expression levels in individual cells.

An important result here is that the rigorous unbiased estimation of $R_{\alpha }^{2}$ can be done based on a time series of normalised measurements of the difference between the means of log-transformed expression levels in isolated cohorts. The normalisation by the value immediately after sorting is a critical part of the inference procedure. A similar normalisation by the initial value was used by Singh et al. [32] to analyse the temporal evolution of the squared coefficient of variation of a single population, under a model that assumed that the observed variation was completely due to noise in gene expression. Using this type of analysis in settings of transcription inhibition, these authors [32] assessed whether noise in mRNA production and degradation or promoter activity fluctuations contribute to noise in protein expression. The normalisation of the differences by their initial value (*t* = 0) in the present work formalises the definition of how much of the initial difference, introduced by the process of sorting by design, remains at later times (function Ω(*t*)). Hence, a key requirement is that the isolated cohorts being compared have different means just after sorting. This strongly argues to using high and low expressors as the basis for quantification, in order to maximise the measurement resolution. In practice, one has to manage a tradeoff between how extreme are the expression levels (to increase resolution and dynamic range of the readout) and how many cells are contained in the cohorts (sample size). One cannot overstate the absolute requirement for an adequate experimental design that guarantees independent measurements of the expression levels to sort the high and low expressors and to quantify their expression means at *t* = 0 and subsequent time points. If the measurement errors of the sorting and of the quantifications are not uncorrelated, the statistical effect of regression to the mean will lead to inaccurate under-estimates of both $R_{\alpha }^{2}$ and *τ*_*T*_. In the analysis of the TCR expression we relied on fresh re-staining of cultured cells and the independent reacquisition of the TCR intensity measurements after sorting. Ideally, different TCR labels (e.g. antibodies to different epitopes) should be used to sort and to quantify the cellular expression. If experimental limitations preclude the independence of sorting and quantification measurements, the effect of regression to the mean should be part of the data analysis.

The estimation based on the mean expression levels broadens the range of applications. It is often argued that the standard experimental techniques that measure bulk expression are obsolete in the context of the studies of gene expression noise, because their population-averaged readouts mask cell heterogeneity (see, for example, [10, 47]). The present analysis framework enables to use these techniques, as one may combine the isolation of cells (the only step requiring the analysis at the single-cell level), with population-averaged readouts to quantify $R_{\alpha }^{2}$ and *τ*_*T*_. The function Δ(*t*), which is at the core of the estimation process, can be approximated as the logarithm of the fold-ratio between the raw mean values of the two populations. In theory, by measuring $R_{\alpha }^{2}$ and the full population variance ($\sigma_{T}^{2}$), one could estimate the actual values of $\sigma_{W}^{2}$ and $\sigma_{\alpha }^{2}$ in Eq 13. This would allow one to compare the values of $\sigma_{W}^{2}$ and $\sigma_{\alpha }^{2}$ in different biological populations. Also, the variances of the isolated cohorts can be further informative, allowing the estimation of the ratio between the absolute values of the contribution of the stable component in the isolated cohort and in the starting population. However, the estimate obtained in this way is biased, under-estimating the true value by up to 20%. Consequently, if an estimate of this ratio is needed, we suggest a simulation-based approach.

Empowered by the quantitative framework, we analysed the variation in the expression levels of the T-cell receptor (TCR) in mouse CD4^+^ T cells. The variation in the expression levels of some membrane receptors of T cells, such as CD5 [48–50] and CD127 [51] shows some stable component, whereas the expression of IL-4 and IL-10 is unstable and volatile [13, 15]. With the increasing availability of single cell genomics, proteomics and metabolomics techniques there is accumulating evidence that T cell populations that hitherto were perceived as homogeneous are in fact complex mixture of cell types and cell states, which may be reversible and transient, raising the issue of stability and dynamics. From a practical perspective, different mouse models are available with different genetic diversity in the TCR loci, which gives a handle to tease apart genetic and non-genetic components of variation. Hence, in our analysis of TCR expression levels we studied a genetically heterogeneous polyclonal population, and also a particular isogenic population, from Marilyn TCR transgenic mouse [27] with *Rag2*-deficient background. These two populations display distinct variances of the TCR levels that are, not surprisingly, positively associated with the genetic TCR diversity. We asked whether these two populations could be described by equal or different values of $R_{\alpha }^{2}$ and *τ*_*T*_ and found that the most parsimonious explanation for the data was a model where only one of these parameters differs. The model with different *τ*_*T*_ and the same value of $R_{\alpha }^{2}$ performed marginally better based on the AIC. However, the point estimate of the characteristic time of the polyclonal population in this scenario was about 20 days, which is unreasonably uncertain given the implied extrapolation beyond the experimental observation time of 96 hours. Furthermore, even if long time scales have been described for the restoration of a bimodal population distribution from selected unimodal cohorts (e.g. over 30 days in [18, 44]), the scenario of very distinct *τ*_*T*_ values for wildtype and transgenic populations is biologically unsound. This scenario requires that the expression of transgenic TCR would differ from natural TCR expression in terms of the protein turnover rate *β* as well as noise in gene expression, such that the same $R_{\alpha }^{2}$ could be present in populations with markedly different variances (Fig 8). Therefore, based on these statistical and biological considerations we rejected this scenario. We concluded in favour of the scenario in which the TCR expression fluctuates with a characteristic time of 32 hours in the both populations, which differ in the values of $R_{\alpha }^{2}$, the polyclonal having $R_{\alpha }^{2}=71\%$ and monoclonal Marilyn having $R_{\alpha }^{2}=17\%$. The relatively small yet not negligible value of $R_{\alpha }^{2}$ obtained for the latter population may be particular and not necessarily generalisable to other TCR-transgenic populations. It is worth mentioning that the analysis of another such TCR transgenic population led to a higher $R_{\alpha }^{2}$ value [52], suggesting that transgenic populations, which are known to display different variance of the TCR levels (e.g. [36]), may also differ in the extent of the stable component of variance.

The capacity to analyse the variation in TCR expression validates experimentally the theoretically-designed methodology. We could quantify the two key parameters in the two cell populations, implying that the methodology has enough power to resolve the stable and unstable components of variance even when the unimodal distribution of interest is remarkably narrow.

Beyond this key methodological result, what do the actual estimates of $R_{\alpha }^{2}$ and *τ*_*T*_ tell about phenotypic variation in TCR expression?

High and low expressors were maintained *in vitro* for as long as possible in the absence of any stimulation thus precluding cell division. Using this setup, we focused on cell-intrinsic components only, and avoiding the above mentioned complications arising from cell division. As a consequence of this choice, the present data do not exclude the possibility that signals arising from the intermittent stimulus from the antigen-presenting cells in the *in vivo* environment may change the values of both $R_{\alpha }^{2}$ or *τ*_*T*_ for the populations tested. Also, cell division is expected to decrease the timescale of the fluctuations in a twofold manner. First, protein dilution into the daughter cells may effectively reduce the value of *β*, even if yeast studies indicate that protein levels are remarkably constant if corrected for cellular volume [53]. Second, cell division may affect the stability of epigenetic modifications facilitating the transitions between chromatin states or bistable transcriptional switches that affect quantitatively TCR expression in this way reducing the effective *τ*_*T*_. A similar point was made in a study [54] of induced pluripotent stem cells. These cells maintained a memory of transcriptional and epigenetic signatures indicative of the cell of origin that vanished with sequential passages. Hanna et al. [55] reported a similar impact of cell division itself. Furthermore, cell division and generation time variability may introduce cell-extrinsic deformations of the expression distribution by differential selection of lineages (see [56] for a theoretical analysis). These potential peculiarities of the experimental design notwithstanding, the estimates of $R_{\alpha }^{2}$ and *τ*_*T*_ are to our knowledge the first reported values and therefore interpreting the meaning of these values requires indirect comparison with other estimates.

The characteristic time of the variation in protein expression represents a transient memory of expression levels [7]. Various studies have quantified the dynamics of fluctuations in expression levels of various molecules, reporting characteristic times that range from hours [7, 12] to days and weeks [14, 16–18, 44]. In studies quantifying the dynamics of the percentage of T cells expressing cytokines [13, 15], the effective timescale was estimated to be about 70 hours for the cytokines IL-10 [13] and IL-4 [15], which was linked to the slow dynamics of chromatin remodelling [13, 15]. The longer time scales were systematically obtained in scenarios with cell division and that involved the restoration of a multimodal distribution of expression levels from biased cohorts. The dynamics of multimodal distributions, in which cells switch between overtly distinct subpopulations, may correspond to transitions between cellular states. These For the unimodal TCR expression, we estimated an effective timescale of 32 hours, in the absence of cell division. This timescale is shorter than that necessary to restore a full multimodal distribution from extremely biased cohorts [18, 44]. The TCR protein complex is arguably one of the most complex receptors in terms of its composition, trafficking and regulation. In quiescent cells, such as the naive cells analysed here, it is continuously recycled between the plasma membrane and intracellular membranes with a fast rate of less than an hour. The TCR in the ensemble of these two pools has a slow turnover rate. The treatment with protein synthesis inhibitor up to 12 hours led only to modest changes in expression levels [57], suggesting that *β* might be greater than 12 hours. However, this estimate is potentially problematic, since this treatment may alter the regulation of the TCR complex levels, as it may up-regulate the expression of the mRNA encoding its *ζ* chain sub-unit [58]. Sousa and Carneiro [59] estimated the baseline TCR turnover in an human T cell line by fitting the dynamics of the mean upon short-term stimulation, and found a value for *β* of 15 hours. Both values [57, 59] are compatible with the effective timescale estimated here, which lumps protein stability and the auto-correlation time of the rate of protein production, and suggest that *β* is of the same order of magnitude as *τ*_*T*_ in the case of the TCR.

**Fig 9:**
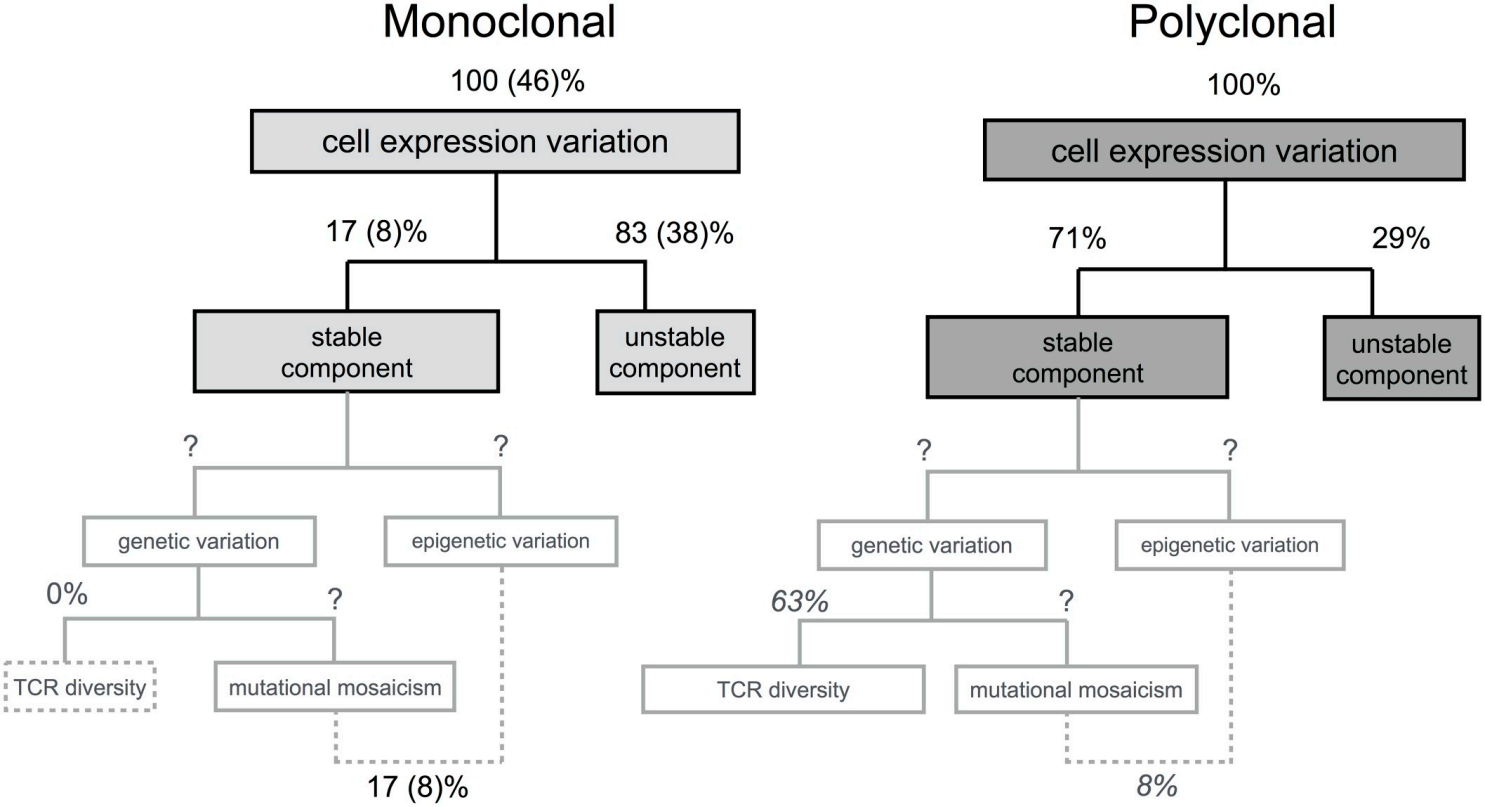
Overview of the components of TCR expression levels variation in naive CD4 T cells from monoclonal Marilyn transgenic and polyclonal wildtype mice. The first partition of the variance in each population corresponds to the stable and unstable components experimentally estimated in this article. The further partitions of the stable component are indicative of the putative genetic and epigenetic causes of the variation. The percentages represent the expected proportions of the variance in log transformed TCR expression levels explained by the indicated components. The values in brackets in the diagram of the monoclonal population were normalised by the variance in the polyclonal population. The values in black are experimental estimations. The values in grey italic are guesses obtained by assuming that the variance explained by somatic chimerism and/or epigenetics is the same in polyclonal and transgenic populations.

The different components that may underly the stable variation in TCR expression levels are systematically addressed in Fig 9. The mean TCR level has been shown to be distributed among the V_*β*_-family subsets in CD4 as well as CD8 human T cell populations [37], under conditions in which there was a strong correlation with cell size. Given that the high and low expressors cohorts in our experiments had virtually the same size distribution as assessed by the respective forward scatter signal, it is likely that part of the stable variance in TCR expression is in fact due to the genetic diversity at the TCR locus. The question is whether genetic diversity ultimately explains all the stable variation. The estimate of a positive value for $R_{\alpha }^{2}$ in the nominally monoclonal TCR transgenic population suggests that non-genetic variation may contribute to stable differences in expression levels of the TCR among cells. This might be a particular feature of TCR-transgenic populations, as their relationship to the actual clones in a polyclonal T cell population is not trivial. The specific mechanism that would mediate such non-genetic variation is unclear at present. We speculate that the stable component in this system may arise from a myriad of chromatin modifications in the form of “molecular switches”, which would affect, directly or indirectly, the expression of the TCR. This speculation is inspired on theoretical studies [13, 60], which predicted these marks to be stable once fully established, but also potentially variable among cells. In terms of the full range of modifications affecting expression of the TCR in *cis* and in *trans*, some could be present, while others could be absent in each individual cell in a stochastic yet stable pattern of modifications [13, 60]. In those cells in which the balance of modifications happens to be tilted towards those inducing expression, levels of the TCR would be higher than average, while in cells with lower TCR levels this balance would be shifted in the direction of those leading to decreased expression. Similar considerations could be made to any epigenetic variants in any of the vast number of transcription factors and regulatory proteins that control the TCR complex expression. Finally, in this enumeration of the causes of stable variation in TCR levels, it is worth noting that despite we have been referring to the population of T cells in Marilyn transgenic mice as “monoclonal” throughout this article, the cells are not a T cell clone derived from a mature T cell. Instead, they are continuously differentiating in the thymus and being exported to circulation. One cannot rule out that these T cells or their bone marrow and thymic precursors underwent sporadic somatic mutations in any of those genes affecting TCR complex expression. The genetic mosaicism of somatic tissues has been well documented (reviewed in [61]) following the advent of single cell sequencing, and thus, one must envisage the possibility that part of the stable variation observed in transgenic TCR expression is due to *bone fide* genetic variation. Simple back of the envelop calculations suggest that epigenetic variants and/or mutational mosaicism outside the TCR locus may represent more than 1/6 of the stable variance in the wild type CD4 T cell. Assuming that epigenetics and/or mosaicism explain the same amount variance in both monoclonal and polyclonal populations, that all the stable variance in the former (which corresponds to 17% relative to monoclonal and 8% relative to polyclonal variances) is explained by these two processes and that the stable variance in the latter is explained by these two processes and by TCR diversity, we have that in the polyclonal population 8/71 of the stable variance is explained by epigenetics/mosaicism and 63/71 is explained by genetic TCR diversity (see Fig 9).

Our quantitative framework makes a connection between systems biology, in particular gene expression noise [9], and quantitative genetics [62]. In both domains, decomposing the variance, or another measure of variation, have been instrumental in studying the properties of different biological systems (see, for example, [42]). The notion of intrinsic and extrinsic noise put forward by Elowitz and colleagues [4, 19] is based on decomposing the coefficient of variation of expression levels into these two noise sources. Others have focused on either generalising this distinction or developing new decompositions [21, 23, 24, 63]. In fact, these approaches may be combined with the framework developed here, to further partition the unstable component, for example, into intrinsic and extrinsic noise. Likewise, in connection with quantitative genetics, parameter $R_{\alpha }^{2}$ can be interpreted as the “heritability” in expression levels of a population. This arises from an analogy with the decomposition of phenotypic variation into a contribution from additive genetic variation and another due to environment (see, for example, [28, 62]), neglecting non-additive genetic variation. At present, this constitutes a mere analogy, since $R_{\alpha }^{2}$ is defined even in the absence of cell division.

Hitherto, the studies on the phenotypic variation in gene expression levels at the individual cell level have relied experimentally on timelapse imaging of single cells or on population snapshots using single-cell resolution techniques such as flow cytometry, qPCR, RNAseq or Cytof. The quantitative framework and methodology proposed here, relying on estimates of the mean of the expression levels in appropriately selected cohorts, enables studying the sources and dynamics of the variation in cellular expression levels by conjugating a single step of sorting with the full gamut of transcriptomic, proteomic and metabolomic technologies available to measure bulk expression. This opens new prospects for studying quantitative traits and responses in heterogeneous cell populations. Furthermore, the model for expression levels considered here can be further extended to incorporate, for example, the gene regulatory networks regulating cell differentiation (*e.g*. [64]). Finally, the sophistication of DNA recording-based methods to account for past fluctuations in the transcript levels of a cell or its lineage will generate massive sets of single-cell transcriptomics data points suitable for decomposition into stable and unstable components [65]. In summary, we have put forward a solid theoretical framework to dissect the components of variation in expression levels according to their stability and dynamics, which enables the further analysis of how different molecular mechanisms may modulate each component.

## Materials and methods

### Ethics statement

This research project was ethically reviewed and approved by the Ethics Committee of the Instituto Gulbenkian de Ciência, and by the Portuguese National Entity that regulates the use of laboratory animals (DGAV—Direção Geral de Alimentação e Veterinária (license reference: 0421/000/000/2013). All experiments conducted on animals followed the Portuguese (Decreto-Lei number 113/2013) and European (Directive 2010/63/EU) legislations, concerning housing, husbandry and animal welfare.

### Notation

The function log(⋅) denotes the natural logarithm, and random variables are represented as bold symbols, as in ***x***. We use E[x] to denote the expected value of a random variable ***x***, and V[x] the variance. The notation z∼LN(μ,σ) represents a random variable ***z*** following a lognormal distribution with parameters *μ* and *σ*, having therefore the probability density function:

$$\begin{array}{c} f(z)=\frac{1}{\sqrt{2\pi }\;\sigma \;z}\text{exp}(-\frac{1}{2\sigma^{2}}\;{\left(\text{log}\;(z)-\mu \right)}^{2}) \end{array}$$ (24)

### Numerical simulations

Simulations of the model based on stochastic differential equations were performed using custom software written in C++, based on the GNU Scientific Library (http://www.gnu.org/software/gsl/). For a given value of the parameters *τ* and *β*, the stochastic model (Eqs 4 and 5) was simulated, using the Brent-Dekker method (GNU Scientific Library) to adjust the value of *σ* so as to obtain the desired value of *σ*_*W*_.

Simulations of cell sorting experiments to isolate appropriate cohorts were done using an initial population with *σ*_*T*_ = 0.3, having 1.2 × 10^6^ cells and 2 × 10^4^ sub-populations, with the number of cells per sub-population following a multinomial distribution. From the starting population, 10% of cells were isolated. As a simple approximation of an experimental setting, each isolated cohort was divided into 3 replicates, and simulated for a given period of time, with snapshots of each replicate being collected at equally spaced times.

### Data analysis, fitting and model selection

Numerical analysis was conducted using MATLAB (Mathworks). The exponential model was fitted to the data by non-linear least squares. To study the relationship between *τ*_*T*_ and parameters *β* and *τ*, simulations were ran for several combinations of values of ($R_{\alpha }^{2}$, *β*, *τ*). The values of *τ*_*T*_ were estimated by fitting the exponential model. Fitting the ensemble of the experimental data was done by equally weighting each experiment, based on the number of data points per experiment. Values of the Akaike Information Criterion (AIC) were corrected for small sample size, as highlighted in section 2.4 of ref. [41], and include the residual variance as an additional effective parameter being estimated for each model. Confidence intervals (95%) were obtained by bootstrapping each experiment separately, then fitting the ensemble of the data.

### Mice

C57BL6/J and B6.*Rag2^−/−^* mice were obtained from the Jackson Laboratory. Marilyn mice [27] were kindly provided by Olivier Lantz (Institut Curie, France), and bred with B6.*Rag2^−/−^* to produce Marilyn.*Rag2^−/−^*. Mice were bred and maintained under specific pathogen free conditions at the animal house of the Instituto Gulbenkian de Ciência, and used for experiments with ages between 8 and 12 weeks.

### Antibodies and flow cytometry

Flow cytometry was performed using a Beckman-Coulter CyAN ADP. Fc receptors were always blocked prior to staining, by incubation with FcBlock (2.4G2, produced in-house). Cells were stained at 4°C, in ice-cold buffer with PBS, 5% fetal bovine serum (PAA), and, except in the case of sorting, with 0.1% sodium azide.

Monoclonal antibodies produced in-house used were: anti-TCR-C*β* (H57-597), anti-CD4 (GK1.5), anti-CD8 (YTS169.4), anti-CD25 (PC61), anti-CD45RB (16A), anti-CD62L (MEL-14), anti-B220 (RA3-6B2), anti-MHC-II (M5/114), anti-Mac1 M1/70), anti-CD3*ϵ* (2C11), anti-CD3*ϵ* (2C11). Commercial antibodies were: anti-CD49b (pan-NK, DX5, BD), anti-CD4 (RM4-5, BD), anti-CD44 (MEL-14, eBioscience), anti-TCR*γδ* (GL3; BD). Biotinylated antibodies were further labeled with PE-Streptavidin (BD).

### Cell sorting and *in vitro* cultures

Single-cell suspensions were prepared from lymph nodes, and also spleens in the case of Marilyn.*Rag2^−/−^* animals (due to limited number of cells), by passing cells through a nylon mesh. Cohorts of cells were sorted according to the TCR levels on a FACSAria (BD), using a strategy based on negative selection of CD4^+^ T cells. Briefly, cells were stained for TCR (anti-TCR-C*β*) and lineage markers not expressed by naive CD4^+^ T cells, and then lineage^-^ cells falling within the desired TCR gates (illustrated in Fig 6 and S1 Text section H) were sorted. A polyclonal naive population was sorted as CD45RB^high^, lineage^-^ (CD8, pan-NK, B220, TCR*γδ* and CD25) cells, while Marilyn cells were sorted as CD62L^+^, lineage^-^ (B220, CD11c, pan-NK, Mac1, MHC-II). The use of CD62L as an alternative marker of naive cells allows for a more efficient sorting (due to a slow loss in the CD45RB signal throughout the sorting), given the limited number of cells, based on the fact that the vast majority of Marilyn cells retain a naive phenotype [27]. Before each sorting for Marilyn cells, the gating for CD62L^+^Lineage^-^ cells, when analysed in a control sample also labeled for CD4, includes more than 80% of TCR^+^CD4^+^ Marilyn cells. Purities of the sorted populations were assessed by staining aliquots of the sorted populations for CD4 expression, were typically greater than 96%.

After sorting, T cells were cultured in flat-bottom 96-well plates (50 × 10^3^ cells per well), in RPMI (Invitrogen), 10% fetal bovine serum (PAA), 1% Sodium Pyruvate (Gibco), penicillin/streptomycin (Gibco), gentamycin (Sigma), 50*μ*M 2-ME (Gibco), in an incubator at 37°C, with 5% CO_2_.

TCR levels were quantified by staining, under optimal, saturating conditions, with anti-TCRC*β* antibody, which binds to the constant region of one of the sub-units of the TCR (see, for example, [66]). Cells maintained in culture were analysed at different time-points by re-staining the TCR, using the same antibody anti-TCRC*β* (clone and fluorochrome) as used for the sorting. In each time-point, 3 replicates (wells) of each sorted population were analysed. The fresh re-staining of the cells is essential to produce measurements of the TCR levels that are independent and uncorrelated with the sorting measurements, eliminating biases expected from the statistical effect of regression to the mean. Also, it avoids additional cell stress and death arising from treatment of cells with acidic buffers to remove antibody bound (for example, [67]). In each experiment, an additional population (control) was sorted in parallel, keeping the same gates used for all expressors, but without staining for the TCR, as a control for the impact of this staining. In each time-point, TCR levels of the control population were compared against those of “all expressors”, confirming that the staining for the sorting does not induce massive changes in TCR expression levels.

Data were analysed using FlowJo 8.8.7 (Tree Star Inc.). Cells gated on forward-scatter and side-scatter, live cells (propidium iodide negative) and CD4^+^ cells. For the analysis of TCR levels, cells were further gated on CD62L^+^ cells, to reduce experimental variation in TCR levels. Percentages of CD62L^-^ cells were always lower than 20% in early time-points (up to 48 hours), and similar to those from control cells, arguing against impact of staining for the TCR in order to sort cells. Gated data was exported as text files and analysed in MATLAB (Mathworks) using custom code.

## Supporting information

S1 TextAll the supporting information is provided in a single document with the following sections: **A**- Detailed derivation of the mean and variance of the full population. **B**- Basic properties of the logarithmic transformation. **C**- Model of protein expression in a cell population for untransformed values. **D**- The asymptotic difference between the means of log-transformed expression levels in two distinct cohorts is given by $R_{\alpha }^{2}$. **E**- Robustness of the equality $\Omega_{H,L}\left(\infty \right)=R_{\alpha }^{2}$ relative to model assumptions. **F**- Dynamics of the mean of log-transformed values. **G**-Analysis of the variances of isolated cohorts. **H**- Forward scatter distributions in sorted cohorts of high and low expressors.(PDF)
