# Supplementary material for: Partitioning stable and unstable expression level variation in cell populations: A theoretical framework and its application to the T cell receptor
Source: PLoS Comput Biol. 2020 Aug 25;16(8):e1007910. doi: 10.1371/journal.pcbi.1007910 (PMC7498022; doi:10.1371/journal.pcbi.1007910)
Supplement: S1 Text — All the supporting information is provided in a single document with the following sections: A- Detailed derivation of the mean and variance of the full population. B- Basic properties of the logarithmic transformation. C- Model of protein expression in a cell population for untransformed values. D- The asymptotic difference between the means of log-transformed expression levels in two distinct cohorts is given by Rα2. E- Robustness of the equality ΩH,L(∞)=Rα2 relative to model assumptions. F- Dynamics of the mean of log-transformed values. G-Analysis of the variances of isolated cohorts. H- Forward scatter distributions in sorted cohorts of high and low expressors. (PDF) [file pcbi.1007910.s001.pdf]

SUPPORTING INFORMATION

Partitioning stable and unstable expression level variation in cell populations: a theoretical framework and its application to the T cell receptor

Thiago S. Guzella[1], Vasco M. Barreto[2,*], Jorge Carneiro[1,*]

1 Instituto Gulbenkian de Ciência, Oeiras, Portugal
2 CEDOC - Chronic Diseases Research Center, NOVA Medical School, Universidade Nova de Lisboa, Lisboa, Portugal

* vasco.barreto@fcm.unl.pt, jcarneir@igc.gulbenkian.pt

A Detailed derivation of the mean and variance of the full population

This section presents the detailed derivation of the mean and variance of the full population, given the parameters describing the sub-populations (equations 2 and 3 in the main text). It is shown that the variance in expression levels of the full population can be decomposed into various terms. Under some reasonable assumptions regarding the correlation between the parameters describing the sub-populations, the decomposition takes the form of the well-known "law of total variance".

A.1 Mean and variance given the parameters of each sub-population

We start from the mixture model formulation, where x represents protein expression levels:

$$f(x \mid \theta_1, \theta_2, \ldots, \theta_N) = \sum_{i=1}^{N} w_i \, f_i(x \mid \zeta_i) \tag{A.1}$$

where $\theta_i = (w_i, \zeta_i)$ are parameters describing each of the N sub-populations. The frequency of the cells in the full population that belong to the i-th sub-population is represented by w_i (see equation 1, main text), while ζ_i parametrizes the probability density function $f_i(x \mid \zeta_i)$ of protein expression levels in that sub-population. For example, in the case that f_i is a normal distribution, ζ_i would be the mean and variance of expression levels in that sub-population.

It follows from equation A.1 that the mean of the full population is given by:

$$\begin{aligned} \mu_F = \mathbb{E}[\boldsymbol{x}] &= \int_{-\infty}^{\infty} x \, f(x \mid \theta_1, \theta_2, \ldots, \theta_N) \, dx \\ &= \sum_{i=1}^{N} w_i \underbrace{\int_{-\infty}^{\infty} x \, f_i(x \mid \zeta_i) \, dx}_{\mu_i} = \sum_{i=1}^{N} w_i \, \mu_i \end{aligned} \tag{A.2}$$

where μ_i is the mean of the i-th sub-population. Therefore, the mean of the full population (μ_F) is simply the average of the means of the sub-populations, weighted by the frequencies w_i. The variance of expression levels, on the other hand, follows from:

$$v_F = \mathbb{V}[\boldsymbol{x}] = \mathbb{E}\left[\boldsymbol{x}^2\right] - \mu_F^2 \tag{A.3}$$

where:

$$\mathbb{E}\left[\boldsymbol{x}^2\right] = \sum_{i=1}^{N} w_i \underbrace{\int_{-\infty}^{\infty} x^2 \, f_i(x \mid \zeta_i) \, dx}_{v_i + \mu_i^2} \tag{A.4}$$

v_i being the variance of each sub-population. Hence, the variance is given by:

$$v_F = \mathbb{V}[\boldsymbol{x}] = \sum_{i=1}^{N} w_i \, \left(v_i + \mu_i^2\right) - \mu_F^2 = \sum_{i=1}^{N} w_i \, v_i + \sum_{i=1}^{N} w_i \, \mu_i^2 - \mu_F^2 \tag{A.5}$$

By Jensen's inequality, it follows that:

$$\sum_{i=1}^{N} w_i \, \mu_i^2 - \mu_F^2 \geq 0 \tag{A.6}$$

and, therefore, the variance v_F is always non-negative, as expected.

Therefore, for the "full" population, one has the mean and variance given by:

$$\mu_F = \mathbb{E}\left[\boldsymbol{x}\right] = \sum_{i=1}^{N} w_i\, \mu_i \tag{A.7}$$

$$v_F = \mathbb{V}\left[\boldsymbol{x}\right] = \sum_{i=1}^{N} w_i\, v_i + \sum_{i=1}^{N} w_i\, \mu_i^2 - \mu_F^2 \tag{A.8}$$

As a remark, these results are independent of the underlying probability density functions f_i describing the expression levels in each sub-population.

A.2 Mean and variance in the limit of large number of sub-populations

In the following, we study the asymptotic properties of the equations describing the mean and variance of expression levels in the full population (equations A.7 and A.8, respectively). In this case, the parameters of the sub-populations introduced in the previous section become themselves random variables, denoted as $\boldsymbol{w}$, for the frequency, $\boldsymbol{\mu}$ as the mean expression level, and $\boldsymbol{v}$ for the variance of a sub-population. To avoid confusing notation, in this section we will refer to the mean and variance of the random variables $\boldsymbol{w}$, $\boldsymbol{\mu}$ and $\boldsymbol{v}$ solely using the notation $\mathbb{E}\left[\cdot\right]$ and $\mathbb{V}\left[\cdot\right]$. In the general case considered here, these random variables are described by a joint density $h(w, \mu, v)$. Hence, the full population to be studied is constructed from a sample $\mathcal{S} = \{(\boldsymbol{w_1}, \boldsymbol{\mu_1}, \boldsymbol{v_1}), \ldots, (\boldsymbol{w_i}, \boldsymbol{\mu_i}, \boldsymbol{v_i}) \ldots, (\boldsymbol{w_N}, \boldsymbol{\mu_N}, \boldsymbol{v_N})\}$ of N vector-valued random variables $(\boldsymbol{w}, \boldsymbol{\mu}, \boldsymbol{v})$ sampled from an unknown distribution. A key simplifying assumption made hereafter is that $(\boldsymbol{w_i}, \boldsymbol{\mu_i}, \boldsymbol{v_i})$ and $(\boldsymbol{w_j}, \boldsymbol{\mu_j}, \boldsymbol{v_j})$ are independent and identically distributed (iid) for all $i \neq j$. In terms of the frequencies, this is not immediate, since w_i and w_j are dependent due to the constraint of unity sum ($\sum_{i=1}^{N} w_i = 1$). However, this dependency is expected to become negligible, as long as the numbers of cells in each sub-population (equation 1, main text) are iid, and N is sufficiently large.

In the following, it is shown that, for a fixed N, the mean and variance of the full population are basically "sample estimates" based on $\mathcal{S}$. Since these estimates are functions of random variables, they are themselves random variables, denoted as $_{\boldsymbol{N}}\boldsymbol{\mu_F}$ and $_{\boldsymbol{N}}\boldsymbol{v_F}$, respectively (as in equations A.7 and A.8):

$$_{\boldsymbol{N}}\boldsymbol{\mu_F} = \sum_{i=1}^{N} \boldsymbol{w_i}\, \boldsymbol{\mu_i} \tag{A.9}$$

$$_{\boldsymbol{N}}\boldsymbol{v_F} = \sum_{i=1}^{N} \boldsymbol{w_i}\, \boldsymbol{v_i} + \sum_{i=1}^{N} \boldsymbol{w_i}\, \boldsymbol{\mu_i}^2 - {_{\boldsymbol{N}}\boldsymbol{\mu_F}}^2 \tag{A.10}$$

It should be highlighted that $\boldsymbol{w_i}$, $\boldsymbol{\mu_i}$ and $\boldsymbol{v_i}$ are, in equations A.9 and A.10, random variables.

In this framework, one is interested in the expected value of the mean and variance of the full population. Under the law of large numbers, the sample estimates (equations A.9 and A.10) will converge to the expected values of the mean and variance for sufficiently large N. We start by deriving the asymptotic mean of the population:

$$\mu_F = \mathbb{E}\left[{_{\boldsymbol{N}}\boldsymbol{\mu_F}}\right] = N\, \mathbb{E}\left[\boldsymbol{w}\, \boldsymbol{\mu}\right] = N\, \left(\mathbb{E}\left[\boldsymbol{w}\right]\, \mathbb{E}\left[\boldsymbol{\mu}\right] + \mathbb{C}\left[\boldsymbol{w}, \boldsymbol{\mu}\right]\right) \tag{A.11}$$

where $\mathbb{C}[\boldsymbol{w}, \boldsymbol{\mu}]$ is the covariance between the random variables $\boldsymbol{w}$ and $\boldsymbol{\mu}$. Given that $\mathbb{E}[\boldsymbol{w}] = 1/N$, by definition of the frequencies, one obtains that:

$$\mu_F = \mathbb{E}[{}_{\boldsymbol{N}}\boldsymbol{\mu_F}] = \mathbb{E}[\boldsymbol{\mu}] + N\,\mathbb{C}[\boldsymbol{w}, \boldsymbol{\mu}] \tag{A.12}$$

Therefore, it follows that, when the $\boldsymbol{w}$ and $\boldsymbol{\mu}$ are uncorrelated, the expected mean of the population is simply the expected mean of the sub-populations, corresponding to equation 2 of the main text.

Following a similar reasoning, one obtains the variance:

$$v_F = \mathbb{E}[{}_{\boldsymbol{N}}\boldsymbol{v_F}] = N\,\left(\mathbb{E}[\boldsymbol{w}\,\boldsymbol{v}] + \mathbb{E}\left[\boldsymbol{w}\,\boldsymbol{\mu^2}\right]\right) - \mathbb{E}\left[{}_{\boldsymbol{N}}\boldsymbol{\mu_F}^2\right] \tag{A.13}$$

where:

$$\mathbb{E}[\boldsymbol{w}\,\boldsymbol{v}] = \frac{1}{N}\,\mathbb{E}[\boldsymbol{v}] + \mathbb{C}[\boldsymbol{w}, \boldsymbol{v}] \tag{A.14}$$

$$\begin{aligned} \mathbb{E}\left[\boldsymbol{w}\,\boldsymbol{\mu^2}\right] &= \frac{1}{N}\,\underbrace{\mathbb{E}\left[\boldsymbol{\mu^2}\right]}_{\mathbb{V}[\boldsymbol{\mu}]+(\mathbb{E}[\boldsymbol{\mu}])^2} + \mathbb{C}\left[\boldsymbol{w}, \boldsymbol{\mu^2}\right] \\ &= \frac{1}{N}\,\left(\mathbb{V}[\boldsymbol{\mu}] + (\mathbb{E}[\boldsymbol{\mu}])^2\right) + \mathbb{C}\left[\boldsymbol{w}, \boldsymbol{\mu^2}\right] \end{aligned} \tag{A.15}$$

Note the appearance of the term $\mathbb{C}\left[\boldsymbol{w}, \boldsymbol{\mu^2}\right]$, containing the additional random variable $\boldsymbol{\mu^2}$. Furthermore, the last term in equation A.13 can be written as:

$$\mathbb{E}\left[{}_{\boldsymbol{N}}\boldsymbol{\mu_F}^2\right] = \mathbb{V}[{}_{\boldsymbol{N}}\boldsymbol{\mu_F}] + (\mathbb{E}[{}_{\boldsymbol{N}}\boldsymbol{\mu_F}])^2 \tag{A.16}$$

which, using equation A.12, becomes:

$$\begin{aligned} \mathbb{E}\left[{}_{\boldsymbol{N}}\boldsymbol{\mu_F}^2\right] &= \mathbb{V}[{}_{\boldsymbol{N}}\boldsymbol{\mu_F}] + (\mathbb{E}[\boldsymbol{\mu}] + N\,\mathbb{C}[\boldsymbol{w}, \boldsymbol{\mu}])^2 \\ &= \mathbb{V}[{}_{\boldsymbol{N}}\boldsymbol{\mu_F}] + (\mathbb{E}[\boldsymbol{\mu}])^2 + 2\,N\,\mathbb{E}[\boldsymbol{\mu}]\;\mathbb{C}[\boldsymbol{w}, \boldsymbol{\mu}] + N^2\,\left(\mathbb{C}[\boldsymbol{w}, \boldsymbol{\mu}]\right)^2 \end{aligned} \tag{A.17}$$

Plugging back equations A.14, A.15 and A.17 into A.13, it follows that the variance is given by:

$$\begin{aligned} v_F = \mathbb{E}[{}_{\boldsymbol{N}}\boldsymbol{v_F}] = \mathbb{E}[\boldsymbol{v}] &+ N\,\mathbb{C}[\boldsymbol{w}, \boldsymbol{v}] \\ &+ \mathbb{V}[\boldsymbol{\mu}] + (\mathbb{E}[\boldsymbol{\mu}])^2 + \mathbb{C}\left[\boldsymbol{w}, \boldsymbol{\mu^2}\right] - \mathbb{V}[{}_{\boldsymbol{N}}\boldsymbol{\mu_F}] \\ &- \left\{(\mathbb{E}[\boldsymbol{\mu}])^2 + 2\,N\,\mathbb{E}[\boldsymbol{\mu}]\;\mathbb{C}[\boldsymbol{w}, \boldsymbol{\mu}] + N^2\,\left(\mathbb{C}[\boldsymbol{w}, \boldsymbol{\mu}]\right)^2\right\} \end{aligned} \tag{A.18}$$

which is reduced to:

$$\begin{aligned} v_F = \mathbb{E}[{}_{\boldsymbol{N}}\boldsymbol{v_F}] = \mathbb{E}[\boldsymbol{v}] &+ \mathbb{V}[\boldsymbol{\mu}] \\ &- \mathbb{V}[{}_{\boldsymbol{N}}\boldsymbol{\mu_F}] + N\left\{\mathbb{C}[\boldsymbol{w}, \boldsymbol{v}] + \mathbb{C}\left[\boldsymbol{w}, \boldsymbol{\mu^2}\right]\right. \\ &\left. - 2\,\mathbb{E}[\boldsymbol{\mu}]\;\mathbb{C}[\boldsymbol{w}, \boldsymbol{\mu}] - N\,\left(\mathbb{C}[\boldsymbol{w}, \boldsymbol{\mu}]\right)^2\right\} \end{aligned} \tag{A.19}$$

The term $\mathbb{V}[{}_{\boldsymbol{N}}\boldsymbol{\mu_F}]$ represents an additional contribution, due to variance in the sample mean of the full population as a consequence of sampling, and tends to zero as N grows. In this case, provided that there is no correlation between the frequencies ($\boldsymbol{w}$) and either the means ($\boldsymbol{\mu}$), the squared means ($\boldsymbol{\mu^2}$) and the variances ($\boldsymbol{v}$) of the sub-population, one obtains equations 2 and 3 of the main text.

B Basic properties of the logarithmic transformation

In this session, we recall some basic properties of the logarithmic transformation. First of all, recall that, a lognormally-distributed random variable $\boldsymbol{x} \sim \mathcal{LN}(\mu, \sigma)$ has expected value, variance and coefficient of variation given, respectively, by:

$$\mathbb{E}[\boldsymbol{x}] = \exp\left(\mu + \frac{1}{2}\sigma^2\right) \tag{B.1}$$

$$\mathbb{V}[\boldsymbol{x}] = \left(\exp\left(\sigma^2\right) - 1\right)\, \exp\left(2\,\mu + \sigma^2\right) \tag{B.2}$$

$$\mathbb{K}[\boldsymbol{x}] = \sqrt{\exp\left(\sigma^2\right) - 1} \tag{B.3}$$

Conversely, the parameters μ and σ of the lognormal distribution are obtained from $\mathbb{E}[\boldsymbol{x}]$ and $\mathbb{V}[\boldsymbol{x}]$ via:

$$\mu = \log\left(\frac{\left(\mathbb{E}[\boldsymbol{x}]\right)^2}{\sqrt{\mathbb{V}[\boldsymbol{x}] + \left(\mathbb{E}[\boldsymbol{x}]\right)^2}}\right) \tag{B.4}$$

$$\sigma = \sqrt{\log\left(1 + \frac{\mathbb{V}[\boldsymbol{x}]}{\left(\mathbb{E}[\boldsymbol{x}]\right)^2}\right)} \tag{B.5}$$

In order to frame the relationship between untransformed and log-transformed values, consider a random variable $\boldsymbol{z}$, and define $\boldsymbol{y} = \log(\boldsymbol{z})$. If $\boldsymbol{y}$ can be well approximated by a normal distribution, then equations B.1 and B.2 can be used to relate the mean and variance of $\boldsymbol{z}$ and $\boldsymbol{y}$:

$$\mu_y \approx \log(\mu_z) - \frac{1}{2}\,\sigma_y^2 \tag{B.6}$$

$$\sigma_y^2 \approx \log\left(k_z^2 + 1\right) \tag{B.7}$$

where $k_z = \mathbb{K}[\boldsymbol{z}]$ is the coefficient of variation of $\boldsymbol{z}$.

C Model of protein expression in a cell population, for untransformed values

C.1 Variation within a sub-population

Starting from equation 4 in the main text, it follows that a population of cells with dynamics of protein expression levels, governed by equations 4 and 5 (main text), has stationary mean given by:

$$\mu = \mathbb{E}\left[\boldsymbol{x_t}\right] = \alpha\,\beta \tag{C.1}$$

and therefore the stationary mean depends on the average expression rate (therefore, α) and on the timescale of protein degradation (β). Moreover, the squared stationary coefficient of variation is given by:

$$k_W^2 = \mathbb{K}\left[\boldsymbol{x_t}\right]^2 = g_k(\exp\left(\sigma^2\right) - 1, \tau/\beta) \tag{C.2}$$

where $g_k(\cdot,\cdot)$ is an arbitrary function, which can be estimated via simulation (analogous to $g(\cdot,\cdot)$ in equation 10 of the main text), and the subscript W highlights that the variation is due to the stochastic process influencing the instantaneous rate of protein expression. Hence, the stationary variance is given by:

$$\mathbb{V}\left[\boldsymbol{x_t}\right] = \left(\alpha\,\beta\right)^2\;k_W^2 \tag{C.3}$$

C.2 Variation among sub-populations

Following equation C.3, the i-th sub-population, with parameter α_i, has mean and variance of protein levels (see equations C.1 and C.3):

$$\mu_i = \alpha_i\,\beta \tag{C.4}$$

$$v_i = \alpha_i^2\;\left(\beta\,k_W\right)^2 \tag{C.5}$$

where it should be noted that k_W^2 is the same for all sub-populations. Applying equations 2 and 3 of the main text, one obtains that the squared coefficient of variation of the full population is given by:

$$k_F^2 = k_W^2 + k_\alpha^2 + \left(k_W\,k_\alpha\right)^2 \tag{C.6}$$

Therefore, equation C.6, based on untransformed values, does not follow the simple additive relationship obtained for the variances of log-transformed values (equation 13 of the main text), given the extra term $\left(k_W\,k_\alpha\right)^2$.

D The asymptotic difference between the means of log-transformed expression levels in two distinct cohorts is given by R_α^2

This section provides an analytic insight into eq. 16 in the main text, which was supported by numerical simulations.

Let $s = log(x)$ be the log-transformed protein expression level in a cell of the full stationary population, at a given time. Based on the mixture model formulation of the full population, s is a stochastic variable such that $s \sim \mathcal{N}(\mu_{log}, \sigma_W^2)$ and $\mu_{log} \sim \mathcal{N}(\mu_{F,log}, \sigma_\alpha^2)$, with μ_{log} and σ_W^2 being respectively the mean and the variance of the log-transformed levels of a stationary sub-population (according to main text equations 9 and 10, respectively), σ_α^2 is the parameter of the lognormal distribution of expression rate α that determines the variance due to the stable component (equation 13), and $\mu_{F,log} = \mathbb{E}[s]$ is the mean of the log-transformed levels of the full population. For convenience, in this section, we will denote the normal probability density of s by $g(s)$.

Let us assume that at, given time $t = 0$, we collect from the stationary full population a single cell with expression level s. What is the mean μ_{log} of the subpopulation to which this cell with expression s belongs to? To answer this we have to consider the probability distribution of μ_{log} given that we selected a random cell with log-expression level s, $p(\mu \mid s)$. We remark that this is formally analogous to the Bayesian inference of the posterior distribution of the mean from a single observation with a normal likelihood with a normal conjugate prior [1]. σ_W^2 plays the role of the known variance of the "observation noise" and μ_{log} is the random parameter with normal prior defined by hyper-parameters $\mu_{F,log}$ and σ_α^2. Under these conditions, we have [1]:

$$\mu_{log} \mid s \sim \mathcal{N}\left(\frac{\sigma_\alpha^2}{\sigma_W^2+\sigma_\alpha^2}s + \frac{\sigma_W^2}{\sigma_W^2+\sigma_\alpha^2}\mu_{F,log}, \left(\frac{1}{\sigma_W^2}+\frac{1}{\sigma_\alpha^2}\right)^{-1}\right) \tag{D.1}$$

The log-transformed expression level of a cell, which had a value s at time $t = 0$, will evolve in time and in the limit $t \to \infty$ will have the expected value:

$$\mathbb{E}[s_{t\to\infty}] = \mathbb{E}[\mu_{log} \mid s] = \underbrace{\frac{\sigma_\alpha^2}{\sigma_W^2+\sigma_\alpha^2}}_{R_\alpha^2} s + \frac{\sigma_W^2}{\sigma_W^2+\sigma_\alpha^2}\mu_{F,log} \tag{D.2}$$

In other words, the expected value of the log-transformed expression level of a randomly selected cell is a linear function of R_α^2, as depicted in Fig 3.

The procedure underlying the analysis in the main text is based on the isolation of two cohorts with log-transformed expression levels respectively $s_A \in A$ and $s_B \in B$, where $A \neq B$. Under these terms, eq. 16 can be rewritten as follows:

$$R_\alpha^2 = \frac{lim_{t\to\infty}\Delta_{A,B}(t)}{\Delta_{A,B}(0)} = \frac{\mathbb{E}[s_A(\infty)] - \mathbb{E}[s_B(\infty)]}{\mathbb{E}[s_A(0)] - \mathbb{E}[s_B(0)]} \tag{D.3}$$

where $s_A(t)$ and $s_B(t)$ are the log-transformed values at time t.

The mean of log-transformed expression levels in the isolated cohort A, at the time of isolation ($t = 0$), is:

$$\mathbb{E}[s_A(0)] = \frac{\int_A s\, g(s)ds}{\int_A g(s)ds} \tag{D.4}$$

Once the dynamics of the expression levels in the cohort A relax to the stationary distribution, the mean value of $s_A(\infty)$ is:

$$\mathbb{E}\left[s_A(\infty)\right] = \frac{\int_A \left(\frac{\sigma_\alpha^2}{\sigma_W^2+\sigma_\alpha^2} s + \frac{\sigma_W^2}{\sigma_W^2+\sigma_\alpha^2} \mu_{F,log}\right) g(s) ds}{\int_A g(s) ds} \tag{D.5}$$

$$= \frac{\int_A \frac{\sigma_\alpha^2}{\sigma_W^2+\sigma_\alpha^2} s\, g(s) ds + \int_A \frac{\sigma_W^2}{\sigma_W^2+\sigma_\alpha^2} \mu_{F,log} g(s) ds}{\int_A g(s) ds} \tag{D.6}$$

$$= \frac{\sigma_\alpha^2}{\sigma_W^2+\sigma_\alpha^2} \frac{\int_A s\, g(s) ds}{\int_A g(s) ds} + \frac{\sigma_W^2}{\sigma_W^2+\sigma_\alpha^2} \mu_{F,log} \tag{D.7}$$

$$= \frac{\sigma_\alpha^2}{\sigma_W^2+\sigma_\alpha^2} \mathbb{E}\left[s_A(0)\right] + \frac{\sigma_W^2}{\sigma_W^2+\sigma_\alpha^2} \mu_{F,log} \tag{D.8}$$

Substituting the expression for $E[s_A(0)]$ and $E[s_A(\infty)]$ for cohort A and corresponding expressions for cohort B on the right hand side of eq. D.1 one obtains:

$$\frac{\mathbb{E}\left[s_A(\infty)\right] - \mathbb{E}\left[s_B(\infty)\right]}{\mathbb{E}\left[s_A(0)\right] - \mathbb{E}\left[s_B(0)\right]} = \tag{D.9}$$

$$= \frac{\left(\frac{\sigma_\alpha^2}{\sigma_W^2+\sigma_\alpha^2} \mathbb{E}\left[s_A(0)\right] + \frac{\sigma_W^2}{\sigma_W^2+\sigma_\alpha^2} \mu_{F,log}\right) - \left(\frac{\sigma_\alpha^2}{\sigma_W^2+\sigma_\alpha^2} \mathbb{E}\left[s_B(0)\right] + \frac{\sigma_W^2}{\sigma_W^2+\sigma_\alpha^2} \mu_{F,log}\right)}{\mathbb{E}\left[s_A(0)\right] - \mathbb{E}\left[s_B(0)\right]} \tag{D.10}$$

$$= \frac{\frac{\sigma_\alpha^2}{\sigma_W^2+\sigma_\alpha^2} \mathbb{E}\left[s_A(0)\right] - \frac{\sigma_\alpha^2}{\sigma_W^2+\sigma_\alpha^2} \mathbb{E}\left[s_B(0)\right]}{\mathbb{E}\left[s_A(0)\right] - \mathbb{E}\left[s_B(0)\right]} \tag{D.11}$$

$$= \frac{\sigma_\alpha^2}{\sigma_W^2+\sigma_\alpha^2} \frac{\mathbb{E}\left[s_A(0)\right] - \mathbb{E}\left[s_B(0)\right]}{\mathbb{E}\left[s_A(0)\right] - \mathbb{E}\left[s_B(0)\right]} \tag{D.12}$$

$$= \frac{\sigma_\alpha^2}{\sigma_W^2+\sigma_\alpha^2} = R_\alpha^2 \tag{D.13}$$

E A preliminary analysis of the robustness of the equality $\Omega_{H,L}(\infty) = R^2_\alpha$ relative to model assumptions

This section addresses whether the equality equation 16 in the main text holds when some of the underlying model assumptions are relaxed. This question deserves a systematic and exhaustive analysis that is beyond the scope of this article. We provided a very preliminary simulation study that indicates that the equality is fairly robust.

In the mixture model of the full population the log-transformed protein expression level $s = log(x)$ is a stochastic variable with $s \sim \mathcal{N}(\mu_s, \sigma_s^2)$, where $\mu_s = \mu_{log}$ is distributed $\mu_s \sim \mathcal{N}(\mu_{F,log}, \sigma_\alpha^2)$, and $\sigma_s = \sigma_W$ is constant and identical for all subpopulations (fig. E.1 A). We asked if equation 16 holds when some of these assumptions are violated in several specific scenarios illustrated in fig. E.1.

We first considered a scenario in which the subpopulation variances were distributed instead of identical for all the subpopulations. We defined $s \sim \mathcal{N}(\mu_s, \sigma_s^2)$, where μ_s and σ_s are independent random variables, $\mu_s \sim \mathcal{N}(\mu_{F,log}, \sigma_\alpha^2)$ and $log(\sigma_s) \sim \mathcal{N}(m, s)$ in one variant and $\sigma_s \sim \texttt{exp}(m)$ in another variant of this scenario (fig. E.1 B).

A second scenario assumed that the subpopulation variance increases with subpopulation mean. We defined $s \sim \mathcal{N}(\mu_s, \sigma_s^2)$ with $\mu_s \sim \mathcal{N}(\mu_{F,log}, \sigma_\alpha^2)$ and σ_s increases linearly with μ_s, i.e. $\sigma_s = s_a + s_b\mu_s$ (fig. E.1 C). In this and in the previous scenario, we defined $\sigma_W = \mathbb{E}[\sigma_s]$.

Finally, we explored a scenario in which the log-transformed expression means of the subpopulations were not normal distributed, as in our model, but followed a shifted gamma distribution . We defined $s \sim \mathcal{N}(\mu_s, \sigma_s^2)$ with $(\mu_s - m_a) \sim \texttt{gamma}(m_b, m_c)$ (fig. E.1 D). As before, $\sigma_s^2 = \sigma_W^2$ is constant and identical in all the subpopulations. m_a, m_b and m_c are the shift, shape and scale parameters that were set to such that $\mathbb{E}[\mu_s] = \mu_{F,log}$ and $\mathbb{V}[\mu_s] = \sigma_\alpha^2$, i.e. such that μ_s has the same mean and variance as in the other scenarios.

For each of these scenarios, we simulated a stationary population of cells indexed i, with $i = 1, ..., N$. Each cell is characterised by the values of the pair of random variables (s_i, μ_{si}), which was drawn randomly from the scenario-specific distributions, fixing the values of $\mu_{F,log}$ and σ_α) and sampling the remaining parameters in a range allowing to explore R^2_α in the interval $[0.0, 1.0]$. To simulate the sorting from the full population of cohorts of high and low expressing cells, we created two subsets L and H such that s_i is lower than the quantile 0.1 for all $i \in L$ and s_i is larger than the quantile 0.9 for all $i \in H$. The asymptotic value of $\Omega_{H,L}(\infty)$ is calculated as:

$$\Omega_{H,L}(\infty) = \frac{\mathbb{E}[\mu_{s i\in H}] - \mathbb{E}[\mu_{s i\in L}]}{\mathbb{E}[s_{i\in H}] - \mathbb{E}[s_{i\in L}]}, \tag{E.1}$$

which is to be compared to $R^2_\alpha = \frac{\sigma_\alpha^2}{\sigma_\alpha^2 + \sigma_W^2} = \frac{\mathbb{V}[\mu_s]}{\mathbb{V}[\mu_s] + \sigma_W^2}$.

The results of simulation of cell population and the scenario of the original model in the main article and the three scenarios that violate its assumptions are illustrated fig. E.1 A-D, as scatter plots (s, μ_s) obtained with parameter sets in which $R^2_\alpha \approx 0.5$, i.e. the stable and unstable variance components of the full population are equally important. The asymmetry of the values of s as well as the dependence of the variance on the values of μ_s is evident in these scatter plots. To summarise how a probability density of s of the full populations, under different scenarios and parameters, compares to a normal probability density, we computed its skewness and kurtosis depicted in fig. E.1 E. The further the skewness and kurtosis of these distributions are from the respective values 0 and 3 characteristic of the normal deviate, the less the simulated population complies with the original model assumptions. On fig. E.1 F, we present for

each scenario and parameter setting the calculated $\Omega_{H,L}(\infty)$ as a function of the R^2_α for each population. In the original model (black symbols) the points lie in the equality line (equation 16). The equality is maintained in the scenario where the variance scales linearly with the mean value of the subpopulations (red symbols). In the scenarios in which the subpopulation variances are log-normal or exponentially distributed the equality breaks down, particularly when the shape of probability density of s differs markedly from the normal distribution (green symbols). A similar depart from the equality is obtained in the scenario in which the subpopulation mean μ_s (i.e. μ_{log}) is gamma distributed, although less marked and only for larger values of R^2_α. Notwithstanding the departs from the line in fig. E.1 F, it is remarkable that equality seems to be rather robust to the violations of the model assumptions explored here.

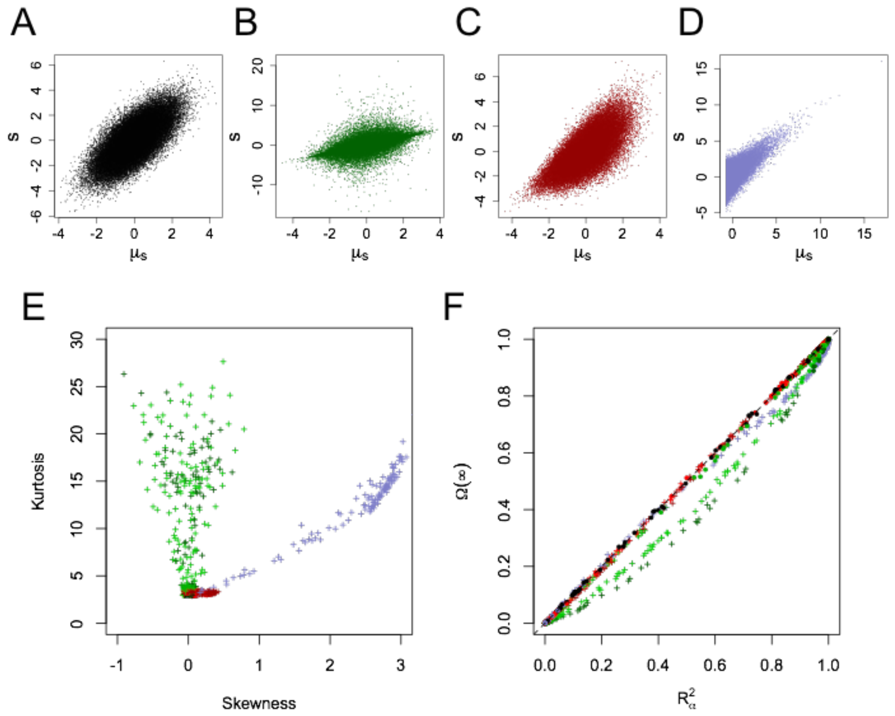

Fig E.1. Robustness of the asymptotic equality $\Omega_{H,L}(\infty) = R^2_\alpha$ to changes in model assumptions under different scenarios. A,B,C,D- Scatter plots of log-transformed expression level s versus the stable mean expression μ_s in each cell of a simulated full population the under the assumptions of the original model (A) and of the three alternative scenarios (B,C, and D). E- Kurtosis and skewness of the stationary distribution of the log-transformed expression levels s for each scenario, exemplified in A-D. F- The relationship between $\Omega_{H,L}(\infty)$ and R^2_α for each scenario. Each point in the two graphs corresponds to a specific scenario and value of σ^2_W. The dots and crossed symbols correspond to scenarios in which the skewness and kurtosis values of the distribution of s were respectively within or outside the 99.9% confidence interval for a normal distribution. The points are coloured according to the scenarios using the same code in all the graphs. The black points represent the original model in the article and the coloured ones represent alternative scenarios. For each scenario, 200 simulations were performed with distinct parameter sets that sample the full range of possible R^2_α values. In all simulations: N=10000 cells, $\mu_{F,log} = 0$, $\sigma^2_\alpha = 1$.

F Dynamics of the mean of log-transformed values

This section studies the dynamics of the log-transformed mean, to provide a rationale for the exponential-like decay of the function $\Delta_{H,L}(t)$ shown in Fig 4 of the main text based on simulations. The first step is the derivation of a linearised approximation of the log-transformed stochastic model that describes protein expression in a sub-population (defined by equations 4 and 5 of the main text). Afterwards, the dynamics of function $\Delta_{H,L}(t)$, which depends on the mean of log-transformed values of high and low expressors, are related to the dynamics of expression levels in the underlying sub-populations.

Since analysis is based on log-transformed values, we define the log-transformed protein level s_t:

$$s_t = \log(x_t) \tag{F.1}$$

such that:

$$x_t = \exp(s_t) \tag{F.2}$$

$$\frac{d\,s_t}{dt} = \frac{1}{x_t}\,\frac{d\,x_t}{dt} \tag{F.3}$$

where x_t is the protein level and y_t is the Ornstein-Uhlenbeck process in the original stochastic model (main text, section **Variation within a sub-population**) Therefore, it follows from equation 4 of the main text that:

$$\begin{aligned}\beta\,ds_t &= \left\{\alpha\,\beta\exp\left(y_t - s_t - \frac{1}{2}\sigma^2\right) - 1\right\}\,dt\\ &= \left\{\exp\left(\log(\alpha\,\beta) + y_t - s_t - \frac{1}{2}\sigma^2\right) - 1\right\}\,dt\end{aligned} \tag{F.4}$$

The dynamics of the mean of log-transformed values in a sub-population are then given by:

$$\beta\,d\,\mathbb{E}[s_t] = \left\{\mathbb{E}\left[\exp\left(\log(\alpha\,\beta) + y_t - s_t - \frac{1}{2}\sigma^2\right)\right] - 1\right\}\,dt \tag{F.5}$$

In order to derive an approximation for the function $\mathbb{E}[s_t]$, it is necessary to simplify the term $\mathbb{E}\left[\exp\left(\log(\alpha\,\beta) + y_t - s_t - \frac{1}{2}\sigma^2\right)\right]$. Introducing:

$$S_t = \log(\alpha\,\beta) + y_t - s_t - \frac{1}{2}\sigma^2 \tag{F.6}$$

and assuming that it is well-concentrated around a certain instantaneous mean, such that it can be approximated by a normal distribution with mean m_t and variance v_t, it follows that (see equation B.1):

$$\mathbb{E}[\exp(S_t)] \approx \exp\left(m_t + \frac{1}{2}\,v_t\right) \tag{F.7}$$

$$m_t = \mathbb{E}[S_t] = \log(\alpha\,\beta) + \mathbb{E}[y_t] - \mathbb{E}[s_t] - \frac{1}{2}\sigma^2 \tag{F.8}$$

$$v_t = \mathbb{V}[S_t] = \mathbb{V}[s_t] + \mathbb{V}[y_t] - 2\,\mathbb{C}[s_t, y_t] \tag{F.9}$$

Assuming that $\left|m_t + \frac{1}{2}\,v_t\right|$ is relatively small, the exponential term in the left-hand side of equation F.7 can be linearized:

$$\mathbb{E}[\exp(S_t)] \approx \mathbb{E}[1 - S_t] = 1 + m_t + \frac{1}{2}\,v_t \tag{F.10}$$

Plugging back into equation F.5, one obtains the following linear approximation for the dynamics of the mean of log-transformed values:

$$\beta \, \frac{d\,\mathbb{E}\left[s_t\right]}{dt} = \log\left(\alpha\,\beta\right) + \mathbb{E}\left[y_t\right] - \mathbb{E}\left[s_t\right] + \frac{1}{2}\left(v_t - \sigma^2\right) \tag{F.11}$$

The equation for the mean of the Ornstein-Uhlenbeck process follows as:

$$\tau \, \frac{d\,\mathbb{E}\left[y_t\right]}{dt} = -\mathbb{E}\left[y_t\right] \tag{F.12}$$

with solution:

$$\mathbb{E}\left[y_t\right] = \mathbb{E}\left[y_0\right]\, \exp\left(-t/\tau\right) = \mu_{y,0}\, \exp\left(-t/\tau\right) \tag{F.13}$$

Therefore, one obtains the following equation for $\mu_t = \mathbb{E}\left[s_t\right]$, which denotes the instantaneous mean of log-transformed values of a single sub-population:

$$\beta \, \frac{d\,\mu_t}{dt} = \log\left(\alpha\,\beta\right) + \mu_{y,0}\, \exp\left(-t/\tau\right) - \mu_t + \frac{1}{2}\left(v_t - \sigma^2\right) \tag{F.14}$$

where recall that v_t (equation F.9) depends on the variances of log-transformed values of the sub-population (s_t) and the Ornstein-Uhlenbeck process variable (y_t), besides the covariance between these two.

In terms of the function $\Delta_{H,L}(t)$, recall that it is defined as (equation 15 of the main text):

$$\Delta_{H,L}(t) = \mu_{H,t} - \mu_{L,t} \tag{F.15}$$

where $\mu_{H,t}$ and $\mu_{L,t}$ are the means of log-transformed values of high and low expressors, respectively, at time t. Using equation A.12, $\Delta_{H,L}(t)$ can be written as:

$$\begin{aligned} \Delta_{H,L}(t) &= \mathbb{E}\left[\boldsymbol{\mu_{H,t}}\right] - \mathbb{E}\left[\boldsymbol{\mu_{L,t}}\right] \\ &\quad + \left(N_h\, \mathbb{C}\left[\boldsymbol{w}, \boldsymbol{\mu_{H,t}}\right] - N_l\, \mathbb{C}\left[\boldsymbol{w}, \boldsymbol{\mu_{L,t}}\right]\right) \end{aligned} \tag{F.16}$$

where $\boldsymbol{\mu_{H,t}}$ and $\boldsymbol{\mu_{L,t}}$ are random variables denoting the instantaneous mean of a particular sub-population in the high (N_h sub-populations) and low expressors (N_l sub-populations), respectively. Neglecting the term of weighted difference between the covariances in equation F.16, it follows that:

$$\Delta_{H,L}(t) \approx \mathbb{E}\left[\boldsymbol{\mu_{H,t}}\right] - \mathbb{E}\left[\boldsymbol{\mu_{L,t}}\right] \tag{F.17}$$

and therefore the dynamics of $\Delta_{H,L}(t)$ can be approximated as:

$$\begin{aligned} \frac{d}{dt}\,\Delta_{H,L}(t) &\approx \frac{d}{dt}\,\mathbb{E}\left[\boldsymbol{\mu_{H,t}}\right] - \frac{d}{dt}\,\mathbb{E}\left[\boldsymbol{\mu_{L,t}}\right] \\ &\mathbb{E}\left[\frac{d}{dt}\,\boldsymbol{\mu_{H,t}}\right] - \mathbb{E}\left[\frac{d}{dt}\,\boldsymbol{\mu_{L,t}}\right] \end{aligned} \tag{F.18}$$

An approximation to the term $\frac{d}{dt}\,\boldsymbol{\mu_{H,t}}$ has been derived in equation F.14, such that:

$$\begin{aligned} \mathbb{E}\left[\frac{d}{dt}\,\boldsymbol{\mu_{H,t}}\right] \approx \frac{1}{\beta} \Bigg\{ &\mathbb{E}\left[\log\left(\boldsymbol{\alpha_H}\,\beta\right)\right] + \mathbb{E}\left[\boldsymbol{\mu_{y_H,0}}\right]\, \exp\left(-t/\tau\right) \\ &- \mathbb{E}\left[\boldsymbol{\mu_{H,t}}\right] + \frac{1}{2}\left(\mathbb{E}\left[\boldsymbol{v_{H,t}}\right] - \sigma^2\right) \Bigg\} \end{aligned} \tag{F.19}$$

and analogously for the low expressors. Plugging into equation F.18, one obtains:

$$\frac{d}{dt}\,\Delta_{H,L}(t) \approx \frac{1}{\beta}\Bigg\{\mathbb{E}\left[\log\left(\boldsymbol{\alpha_H}\right)\right] - \mathbb{E}\left[\log\left(\boldsymbol{\alpha_L}\right)\right] + \left(\mathbb{E}\left[\boldsymbol{\mu_{y_H,0}}\right] - \mathbb{E}\left[\boldsymbol{\mu_{y_L,0}}\right]\right)\exp\left(-t/\tau\right) - \left(\mathbb{E}\left[\boldsymbol{\mu_{H,t}}\right] - \mathbb{E}\left[\boldsymbol{\mu_{L,t}}\right]\right) + \frac{1}{2}\left(\mathbb{E}\left[\boldsymbol{v_{H,t}}\right] - \mathbb{E}\left[\boldsymbol{v_{L,t}}\right]\right)\Bigg\} \tag{F.20}$$

By symmetry, the difference $\mathbb{E}\left[\boldsymbol{v_{H,t}}\right] - \mathbb{E}\left[\boldsymbol{v_{L,t}}\right]$ in equation F.20 is expected to be close to zero. Defining the constants:

$$\Gamma = \mathbb{E}\left[\log\left(\boldsymbol{\alpha_H}\right)\right] - \mathbb{E}\left[\log\left(\boldsymbol{\alpha_L}\right)\right] \tag{F.21}$$

$$\Lambda = \mathbb{E}\left[\boldsymbol{\mu_{y_H,0}}\right] - \mathbb{E}\left[\boldsymbol{\mu_{y_L,0}}\right] \tag{F.22}$$

equation F.20 is simplified to take the form:

$$\beta\,\frac{d}{dt}\,\Delta_{H,L}(t) \approx \Gamma + \Lambda\,\exp\left(-t/\tau\right) - \left(\mathbb{E}\left[\boldsymbol{\mu_{H,t}}\right] - \mathbb{E}\left[\boldsymbol{\mu_{L,t}}\right]\right) \tag{F.23}$$

Finally, using the original approximation in equation F.17, it follows that:

$$\beta\,\frac{d}{dt}\,\Delta_{H,L}(t) \approx \Gamma + \Lambda\,\exp\left(-t/\tau\right) - \Delta_{H,L}(t) \tag{F.24}$$

Introducing the auxiliary variable $U(t) = \Lambda\,\exp\left(-t/\tau\right)$, then equation F.24 can be written as a two-dimensional linear dynamical system:

$$\frac{d}{dt}\begin{bmatrix}\Delta_{H,L}(t)\\ U(t)\end{bmatrix} = \underbrace{\begin{bmatrix}-\frac{1}{\beta} & \frac{1}{\beta}\\ 0 & -\frac{1}{\tau}\end{bmatrix}}_{A}\begin{bmatrix}\Delta_{H,L}(t)\\ U(t)\end{bmatrix} + \underbrace{\begin{bmatrix}\frac{\Gamma}{\beta}\\ 0\end{bmatrix}}_{B} \tag{F.25}$$

subject to the initial condition:

$$\begin{bmatrix}\Delta_{H,L}(0)\\ U(0)\end{bmatrix} = \begin{bmatrix}\delta_0\\ \Lambda\end{bmatrix} \tag{F.26}$$

Since the matrix A (equation F.25) has always non-imaginary eigenvalues, it follows that $\Delta_{H,L}(t)$ is the combination of two exponential decays with mean times given by τ and β and a linear component from B. In general, $\Delta_{H,L}(t)$ is a monotonous decreasing function that tends to Γ and has an inflection point more or less close to the $t = 0$. For the practical purposes of estimating a mean relaxation time, this function can be reasonably approximated by single exponential with a mean time $\tau_T \approx \tau + \beta$, consistently with numerical simulations of the full system depicted in Fig 4 of the main text. In the extreme cases, $\beta \gg \tau$ or $\beta \ll \tau$ the function simplifies to a single exponential decay with the dominant mean time. In the specific intermediate case of $\beta = \tau$ one obtains the solution:

$$\Delta_{H,L}(t) = \Gamma + (\delta_0 - \Gamma)exp(-t/\tau)\left(1 + \frac{t}{\tau}\right), \tag{F.27}$$

in which the linear term $1/\tau$ slows the dynamics which is approximated by single exponential decay with mean time of about 2τ.

G Analysis of the variances of isolated cohorts

In this section, it is shown that analysing the variances of isolated cohorts can provide additional information, given the estimate of R^2_α. This analysis is based on the same simulation setting considered in main text (section **Estimating the relative contribution of the stable component**). However, it was found that the estimates derived herein are much more sensitive to sampling effects. Therefore, the starting populations considered here had a much larger number of cells (see Simulations, subsection G.1).

Fig G.1 shows the variance of log-transformed values as a function of time for the "high expressors". As in section **Estimating the relative contribution of the stable component**, the "all expressors" are also included, as a reference of the starting population. The variance of high expressors is lower than that of the starting population, and either remains constant or increases as a function of time. The same takes place for the low expressors, since the variance is a moment of even order. Finally, as observed for the mean of log values, the asymptotic (stationary) variance is equal to that of the "all expressors" for $R^2_\alpha = 0$, since in this case the unstable component is the only contribution present.

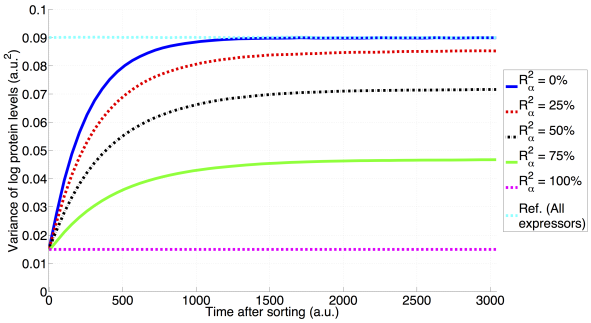

Fig G.1. Variance (log values) of "high expressors" after isolation as 10% of starting populations with different values of R^2_α, but constant σ^2_T. Parameters: $\tau = 500$, $\beta = 5$ and $\sigma_T = 0.3$.

Focusing on the asymptotic (stationary) variance, Fig G.2 shows that the simple, linear, relationship between the mean and R^2_α does not hold in this case. In particular, for $R^2_\alpha \leq 30\%$, the variance of high and low expressors is very close to that of the all expressors. In order to understand the basis for the relationship showed in Fig G.2, we consider hereafter the partitioning of the variance of each isolated cohort, in contrast to the main text, which focused on the starting population. However, the value of R^2_α considered will always refer to that of the starting population.

Recall that the variance of the starting population is given by:

$$\sigma^2_T = \sigma^2_W + \sigma^2_\alpha \tag{G.1}$$

For a general isolated cohort D, based on equation A.19 (section A), the variance is partitioned as:

$$\sigma^2_{T,D}(t) = \sigma^2_{W,D}(t) + \sigma^2_{\alpha,D} + \delta_{T,D} \tag{G.2}$$

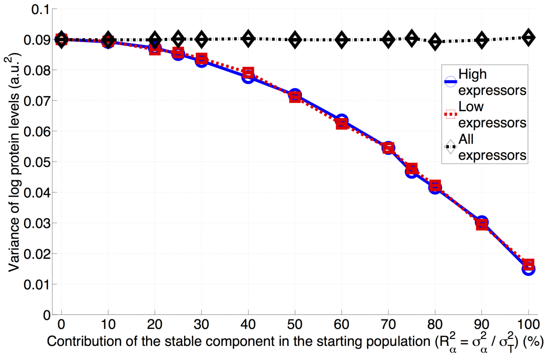

Fig G.2. Asymptotic (stationary) variance of expression levels (log values) of high and low expressors, isolated in the simulations as 10% of the starting population, and also of all expressors. The symbols represent the values obtained from the simulations, while the lines represent linear interpolation. Parameters: $\tau = 500$, $\beta = 5$ and $\sigma_T = 0.3$.

The subscripts H, L and A in place of D will be used to refer to the isolated cohorts corresponding to high, low and all expressors, respectively. The notation $\sigma^2_{W,D}(t)$ highlights that the variance due to the unstable component becomes a function of time, which will increase until the population becomes stationary. The variance due to the stable component in the isolated cohorts is represented by $\sigma^2_{\alpha,D}$, to highlight the fact that it may be different from that of the starting population (σ^2_α) as a consequence of isolating only some sub-populations (see discussion in main text, section **The dynamics of the expression distribution of isolated cell cohorts depends on the relative contribution of the stable component**). Finally, the term $\delta_{T,D}$ in equation G.2 represents a "residual contribution", which may be introduced by the process of isolating cells. It arises from the covariance terms, which may become non-negligible even for a starting population satisfying equation G.1. Note that, by definition, $\delta_{T,A} = 0$ (since the "all expressors" satisfy equation G.1).

In analogy with the standard F-statistic used for comparing the variances of two samples, we will denote by F_D the ratio between the asymptotic variance of the isolated cohort (equation G.1) and the variance of the starting population (G.2):

$$F_D = \lim_{t\to\infty} \frac{\sigma^2_{T,D}(t)}{\sigma^2_T} \tag{G.3}$$

highlighting that this ratio depends only on measurable properties of the two populations. Moreover, define Φ_D as the ratio between the absolute variances of the stable component in the isolated and starting populations:

$$\Phi_D = \frac{\sigma^2_{\alpha,D}}{\sigma^2_\alpha},\; R^2_\alpha \neq 0 \tag{G.4}$$

to denote the relative change, as a consequence of isolating cells, in the variance of the stable component in the "new" (isolated) population. In the following, it is shown that F_D and R^2_α can be used to construct an estimator for Φ_D, denoted as $\widehat{\Phi}_D$. The requirement for $R^2_\alpha \neq 0$ stems from the constraint of $\sigma^2_\alpha \neq 0$.

It follows from taking the ratio between equations G.2 and G.1 that:

$$\begin{aligned} F_D &= \frac{\sigma_W^2}{\sigma_W^2 + \sigma_\alpha^2} + \frac{\sigma_{\alpha,D}^2 + \delta_{T,D}}{\sigma_W^2 + \sigma_\alpha^2} \\ &= \frac{\sigma_W^2}{\sigma_W^2 + \sigma_\alpha^2} + \frac{\left(\sigma_{\alpha,D}^2 + \delta_{T,D}\right)/\sigma_\alpha^2}{1 + (\sigma_W^2/\sigma_\alpha^2)} \\ &= \frac{\sigma_W^2}{\sigma_W^2 + \sigma_\alpha^2} + \frac{1}{1 + (\sigma_W^2/\sigma_\alpha^2)} \left(\Phi_D + \epsilon_{V,D}\right), \; R_\alpha^2 \neq 0 \end{aligned} \tag{G.5}$$

where $\epsilon_{V,D}$ is defined as:

$$\epsilon_{V,D} = \frac{\delta_{T,D}}{\sigma_\alpha^2} \tag{G.6}$$

Using the definition of R_α^2:

$$\Phi_D = \underbrace{1 - \frac{1}{R_\alpha^2}\left(1 - F_D\right)}_{\widehat{\Phi}_D} - \epsilon_{V,D}, \; R_\alpha^2 \neq 0 \tag{G.7}$$

it follows that one estimator for Φ_D, denoted as $\widehat{\Phi}_D$, can be obtained via:

$$\widehat{\Phi}_D = 1 - \frac{1}{R_\alpha^2}\left(1 - F_D\right) \tag{G.8}$$

Hence, the "true" and estimated values are related via:

$$\widehat{\Phi}_D = \Phi_D + \epsilon_{V,D}, \; R_\alpha^2 \neq 0 \tag{G.9}$$

in which $\epsilon_{V,D}$ becomes the bias in the estimation of Φ_D.

Hereafter, we conduct a more detailed analysis of the contributions to the variance in the isolated cohorts, to evaluate the use of the estimator $\widehat{\Phi}_D$ in quantifying Φ_D. This analysis is based on isolating the populations of interest, and simulating until they become stationary. At this point, using the underlying structure of each isolated cohort, the expression levels and number of cells in each sub-population were determined. Using equation A.19, the different terms were then calculated.

To understand the basis of the residual contribution ($\delta_{T,D}$), Fig G.3 depicts the contributions to the asymptotic variance ($\sigma_{T,D}^2$, $\sigma_{W,D}^2$, $\sigma_{\alpha,D}^2$ and $\sigma_{W,D}^2 + \sigma_{\alpha,D}^2$) of each of the isolated cohorts, as a function of the value of R_α^2. For each isolated cohort, the total variance ($\sigma_{T,D}^2$) corresponds exactly to the data shown in Fig G.2, while the term due to the unstable component ($\sigma_{W,D}^2$), being equal to that in the starting population (σ_W^2), is simply $\sigma_T^2\left(1 - R_\alpha^2\right)$. The converse holds for the term arising due to the stable component in the population of all expressors ($\sigma_{\alpha,A}^2 = \sigma_T^2 R_\alpha^2$), since this population is equivalent to the starting one. On the other hand, for high and low expressors, the value of $\sigma_{\alpha,D}^2$, follows a more complicated dependence on R_α^2, reaching a maximum for values of R_α^2 around 60%. Moreover, in these two isolated cohorts, the sum $\sigma_{W,D}^2 + \sigma_{\alpha,D}^2$ is greater than the total variance $\sigma_{T,D}^2$, especially for intermediate values of R_α^2. This difference corresponds exactly to the residual component $\delta_{T,D}$. Moreover, as shown in Fig G.3 (bottom right), it constitutes up to 15% of the total variance, having negative values for all R_α^2. The occurrence of negative values is not unexpected, given that there are both positive and negative terms in equation A.19.

However, it is important to recall that the bias $\epsilon_{V,D}$ in the estimation of Φ_D corresponds to the residual component divided by σ_α^2. The bias is shown in Fig G.4 as a function of R_α^2, keeping in mind that it is only defined for $R_\alpha^2 \neq 0$. While the residual

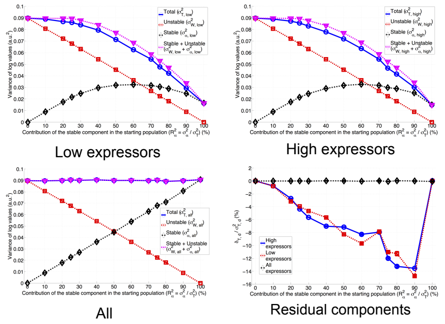

Fig G.3. Properties of the isolated cohorts for various values of R_α^2, always considering log-transformed expression levels. Shown here are the variance components in the various isolated cohorts, along with the residual component ($\delta_{T,D}$). The latter has been calculated based on equation A.19, and the values shown are normalized by the total variance.

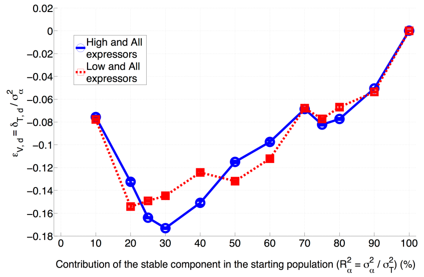

Fig G.4. Bias term $\epsilon_{V,D}$ as a function of R_α^2 (for $R_\alpha^2 \neq 0$), considering the analysis based on the pairs (high, all) and (low, all), determined based on the residual variance (equation A.19) and σ_α^2.

variance has an absolute value of up to 5% (Fig G.3, bottom right), it follows that the bias $\epsilon_{V,D}$ varies from -0.18 to 0, vanishing only for $R^2_\alpha \to 100\%$. Hence, it is expected that $\widehat{\Phi}_D$ under-estimates Φ_D.

Finally, the "true" and the estimated values of Φ_D are shown in Fig G.5. In this figure, Φ_D was calculated based on data on the sub-populations composing each isolated cohort, while the estimate $\widehat{\Phi}_D$ was obtained based on equation G.8. These two values are clearly different for values of R^2_α lower than 70%. Fig G.5 also shows that, when the bias $\epsilon_{V,D}$ is accounted for (using equation A.19, to obtain the residual component and σ^2_α), subtracting it from the estimate results in the "true" value. However, given that the bias cannot be estimated in practice, since it depends on the underlying structure of each cell population, it follows that the estimation of Φ_D via $\widehat{\Phi}_D$ is, indeed, biased in most of the cases.

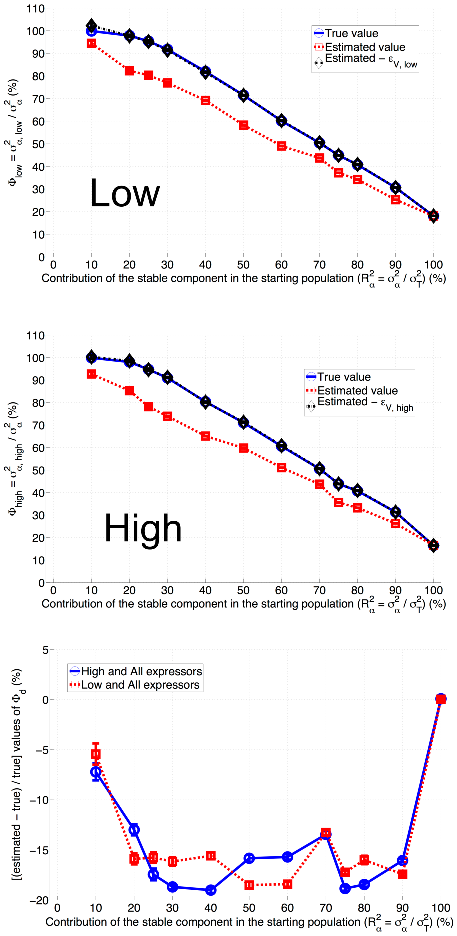

Fig G.5. Comparison between the "true" value of Φ_D and the estimated value $\widehat{\Phi}_D$ obtained according to equation G.8. Each graph also includes the results of subtracting the bias (obtained as in Fig G.4) from the estimated value, to show that it explains the discrepancy between Φ_D and $\widehat{\Phi}_D$.

Therefore, we conclude that the variance can provide additional information. The variance allows to estimate the ratio between the stable component in an isolated cohort (such as the high or low expressors) and the stable component in the starting population

(or its proxy the all expressor cohort). This estimate depends on the value of R^2_α, which can be estimated using the approach outlined in the main text (section **Estimating the relative contribution of the stable component**), and the ratio between the total variances of the two populations being compared (either high and all expressors, or low and all expressors). However, it was shown here that this estimate is biased, due to introduction of a residual component as a consequence of isolating cells based on the expression levels. This bias results typically in an under-estimation of the "true" value of Φ_D by up to 20% of the true value. Hence, we interpret these results to imply that the asymptotic (stationary) variance is uninformative. Further highlighting the approach based on the means to estimate R^2_α, in the case that an estimate of Φ_D is needed, a simulation-based approach is suggested:

1. estimate R^2_α using the analysis of the means
2. using R^2_α, simulate the actual isolation of cells from the starting population, and determine Φ_D

G.1 Simulations

Simulations were done with the same parameters as those reported on the main text (section **Estimating the relative contribution of the stable component**) but with a much larger number of cells in the starting population (30×10^6), and the same number (2×10^4) of sub-populations. Statistics on the sub-populations were calculated based on the asymptotic mean and variance defined by the parameters $\{\alpha_i, \beta, \sigma^2_W\}$ describing each sub-population.

H Forward scatter distributions in sorted cohorts of high and low expressors

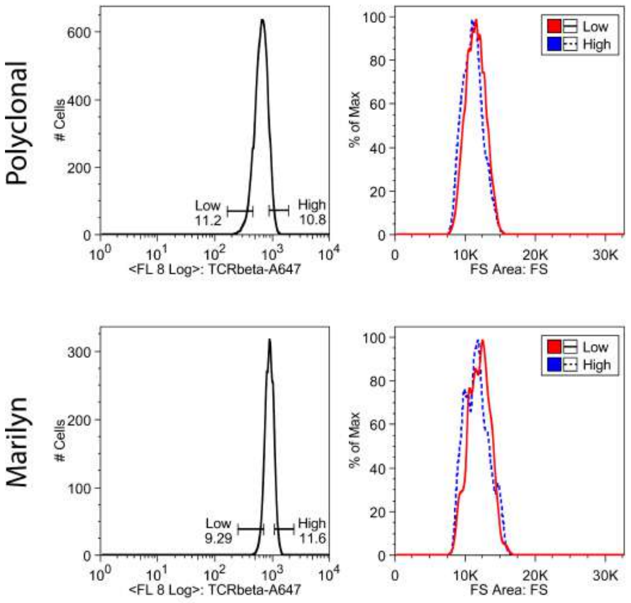

Fig H.1. Forward scatter distributions in cohorts of high and low TCR expressors sorted from naive polyclonal (top) and Marilyn monoclonal (bottom) CD4 T cells. Left: Histograms of TCR intensity in the cell populations are shown on the left together with the gates used to sorting the high and low expressor cohorts. The numbers are the percentage of cells in each cohort. Right: Histograms of Forward Scatter Area for the high (blue) and low (red) expressors immediately after sorting.

References

1. Gelman A, Carlin JB, Stern HS, Rubin DB. Bayesian Data Analysis. 2nd ed. CRC Press; 2003.
